# Development and validation of a Cog‐Free risk predicting tool for dementia in a community setting

**DOI:** 10.1002/gps3.70028

**Published:** 2026-06-01

**Authors:** Jiwen Che, Na Liu, Guirong Cheng, Lu Liu, Yu Luo, Juan Zhou, Ming Chen, Wen Zhou, Dan Liu, Feifei Hu, Xinyan Xie, Xiaochang Liu, Yuanyuan Peng, Yueyi Zhang, Zhiming Wang, Congxia Li, Heqianxi Dong, Chenying Zhang, Wei Tan, Yan Zeng

**Affiliations:** ^1^ Geriatric Hospital Affiliated to Wuhan University of Science and Technology Wuhan Hubei China; ^2^ Hubei Provincial Clinical Research Center for Alzheimer’s Disease Tian You Hospital School of Medicine Wuhan University of Science and Technology Wuhan Hubei China; ^3^ Wuhan Asia Heart Hospital School of Medicine Wuhan University of Science and Technology Wuhan Hubei China

**Keywords:** cognition, cohort studies, community health services, follow‐up studies

## Abstract

**Background:**

Dementia poses a growing global public health burden, particularly in low‐ and middle‐income countries where cognitive screening coverage remains limited. Current risk estimation tools often depend on cognitive testing or biomarkers, restricting their applicability in community and primary care settings.

**Aims:**

To establish and validate a data‐driven analytical framework for developing a cognitive‐testing‐free dementia risk estimation tool (Cog‐Free) using routinely collected health examination data.

**Methods:**

For this prospective cohort study, we developed Cog‐Free, an internet‐based dementia risk estimation tool, using 38 Least Absolute Shrinkage and Selection Operator‐selected risk‐associated variables and the optimal machine‐learning algorithm (logistic regression). The optimal algorithm was internally validated with bootstrap resampling and externally tested in the Chinese Longitudinal Healthy Longevity Survey cohort. The tool was trained and internally validated in 2962 dementia‐free adults aged ≥ 65 years (2018–2024), and its performance was compared with three established cognitive‐testing‐free tools.

**Results:**

Cog‐Free achieved the highest area under the receiver operating characteristics curve in the internal validation set (0.86 [95% confidence interval (CI) 0.82–0.89]) with an accuracy of 0.81 (95% CI 0.78–0.83), sensitivity 0.78 (95% CI 0.68–0.85) and specificity 0.81 (95% CI 0.78–0.84), significantly outperforming three existing tools (DeLong's test, *p* < 0.001). Several previously under‐recognized risk‐associated variables for dementia were identified, such as right‐hand grip strength, cognitive activity, having worse memory than peers, nighttime awakenings and income satisfaction.

**Conclusions:**

Cog‐Free provides a data‐driven, cognitive‐testing‐free and easily accessible approach for early dementia risk screening using routine health data. Its performance and web‐based design suggest potential utility as a pre‐screening and risk stratification tool within community health systems, including settings with limited access to cognitive testing.

## Abstract (highlights)

**WHAT IS ALREADY KNOWN ON THIS TOPIC**

- Existing dementia risk indices, such as Australian National University Alzheimer's Disease Risk Index, Lifestyle for Brain Health and Cognitive Health and Dementia Risk Index, were primarily constructed through expert consensus or meta‐analytic evidence and rely on predefined variables or cognitive testing. These approaches have limited adaptability to low‐ and middle‐income populations and are less suitable for large‐scale routine screening in community settings.

**WHAT THIS STUDY ADDS**

- This study introduces and externally validates a data‐driven, explainable machine‐learning framework for cognitive‐testing‐free dementia risk estimation using routine health examination data. The framework integrates variable selection, multi‐algorithm benchmarking and interpretability analysis, yielding the Cognition‐Free model with strong discrimination (area under the receiver operating characteristics curve = 0.86) and revealing novel, accessible risk‐associated variables, such as grip strength, subjective memory comparison, income satisfaction and defecation timing.

**HOW THIS STUDY MIGHT AFFECT RESEARCH, PRACTICE OR POLICY**

- This analytical framework demonstrates a transparent and reproducible pathway for building practical low‐cost dementia risk models based on routinely collected health data. The web‐based Cog‐Free tool exemplifies how this method can be implemented in primary care to enable scalable early screening, guide targeted prevention and inform public health strategies in resource‐limited regions.

## INTRODUCTION

Dementia represents a major global health challenge amid accelerating population ageing.^1^, ^2^ Early risk estimation and targeted interventions at the stages of subjective cognitive decline or mild cognitive impairment (MCI)^3^ can slow age‐related cognitive decline and delay dementia progression. These measures may also reduce healthcare costs, especially in low‐ and middle‐income countries such as China, where dementia risk is higher than in developed nations.^4^, ^5^ However, cognitive screening among community‐dwelling older adults remains severely insufficient. This is mainly due to a shortage of trained professionals and low willingness among older adults to undergo cognitive testing, partly because of limited awareness of dementia risk.^6^

Multiple factors, such as sociodemographic indicators (age, sex, education, etc.),^1^, ^7^, ^8^ lifestyle factors (physical activity, cognitive activity, smoking, alcohol consumption, etc.),^9^, ^10^, ^11^ physical measures (grip strength, waistline, etc.),^12^, ^13^ comorbidities (hypertension, diabetes, stroke, etc.)^14^, ^15^, ^16^, ^17^, ^18^ and emotional disorders (insomnia, depression, etc.),^19^, ^20^ have been established as closely linked to dementia risk. Can these routinely collected health examination variables be used to estimate dementia risk accurately?

Over the past decade, several dementia risk estimation tools have been developed and validated to profile modifiable risk and protective factors in individuals and populations.^21^, ^22^, ^23^, ^24^, ^25^ Although widely used in research, these tools are seldom implemented in real‐world settings. Models such as the Australian National University Alzheimer's Disease Risk Index (ANU‐ADRI),^21^ the Cardiovascular Risk Factors, Aging, and Dementia Study (CAIDE),^22^ the Lifestyle for Brain Health (LIBRA) index and its modified version (Modified‐LIBRA)^23^, ^24^ and the recently developed Cognitive Health and Dementia Risk Index (CogDrisk)^25^ incorporate evidence‐based risk factors (e.g., education, smoking, diet, depression, etc.) through systematic reviews and meta‐analyses, support self‐administered questionnaires and assign risk scores. However, as they were developed primarily in high‐income countries, their estimative factors may not generalise well to developing nations,^26^ affecting variable weighting and estimative accuracy. Although several models have been developed using data from low‐ and middle‐income countries,^27^, ^28^ they often focus exclusively on urban or rural^28^ populations, limiting their generalisability. Furthermore, some models incorporate biomarkers^29^, ^30^, ^31^ that are not readily available in primary care or routine health examinations, hindering their adoption in frontline primary care settings. This underscores the need to develop and validate a cognitive‐testing‐free risk estimation tool that is broadly representative. In this context, machine learning approaches^32^, ^33^, ^34^ offer flexible and data‐driven methods for integrating multidimensional risk‐associated variables to improve estimation accuracy. Beyond developing a practical tool, there remains a methodological gap in how to systematically integrate multidimensional health data, compare machine‐learning algorithms and ensure model interpretability for dementia risk estimation.

### Contributions

This study introduces a new analytical framework for developing cognitive‐testing‐free dementia risk tools. The framework integrates data‐driven variable selection, algorithm benchmarking and model interpretability assessment, providing a reproducible pathway for community‐level dementia risk modelling. The tool was compared with leading existing tools, including CogDrisk, ANU‐ADRI and Modified‐LIBRA. Such a tool would provide a low‐cost scalable solution for early dementia screening in resource‐limited regions, thereby improving early detection. To our knowledge, this study represents one of the first data‐driven, cognitive‐testing‐free dementia risk tools that systematically evaluate multiple machine‐learning algorithms and directly compare their performance with established screening instruments, suggesting its generalisability and practical value.

## METHODS

**FIGURE 1:**
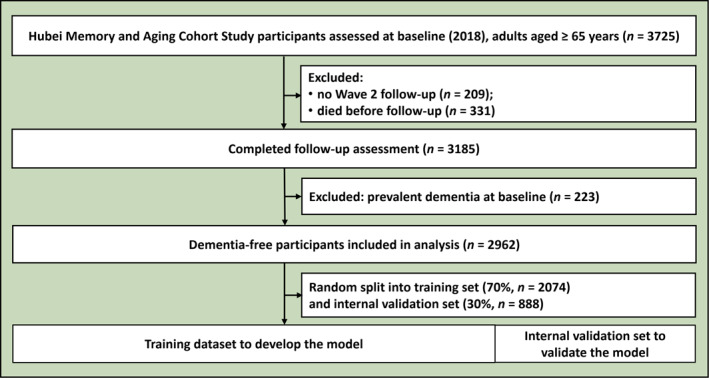
Study enrolment flowchart for dementia risk estimation model development and internal validation.

**FIGURE 2:**
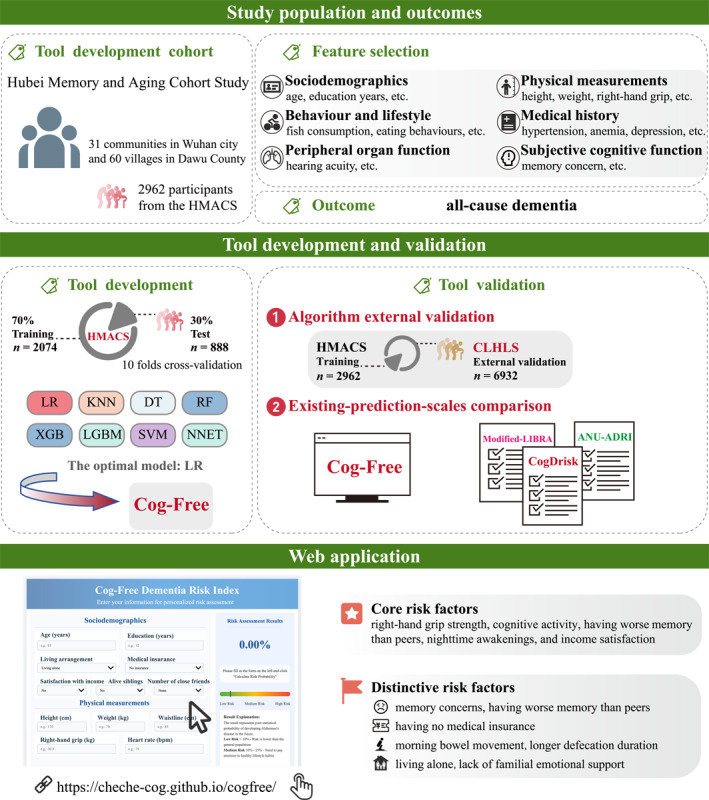
The flowchart for developing and validating Cog‐Free. ANU‐ADRI, Australian National University Alzheimer's Disease Risk Index; CLHLS, Chinese Longitudinal Healthy Longevity Survey; CogDrisk, Cognitive Health and Dementia Risk Index; Cog‐Free, cognition‐free model; DT, decision tree; HMACS, Hubei Memory and Aging Cohort Study; KNN, K‐nearest neighbors; LGBM, light gradient boosting machine; LIBRA, Lifestyle for Brain Health; LR, logistic regression; NNET, artificial neural network; RF, random forest; SVM, support vector machine; XGB, extreme gradient boosting.

The study workflow, including data sources, participants, risk‐associated variable selection, algorithm confirmation, comparison with existing risk estimation tools and a web application, is illustrated in figures 1 and 2.

### Study design and population

This prospective cohort study included participants aged 65 years and older from the Hubei Memory and Aging Cohort Study (HMACS) who were dementia‐free at baseline (2018) and were followed until 2024. The HMACS enrolled community‐dwelling adults using cluster sampling from 31 communities in Wuhan city and 60 villages in Dawu County in 2016, which represents both urban and rural older populations in central China.^3^, ^35^, ^36^ We included dementia‐free participants with at least one follow‐up assessment within 1.5–3 years, and excluded those with severe psychiatric or neurological disorders, and those using cognition‐impairing medications.

### Study procedure

The research team and interviewers, including community hospital physicians, nurses and medical graduate students, received a comprehensive 2‐week training program with a final certification test. Multiple quality control measures, including the use of checklists and follow‐up procedures, were implemented to ensure the accuracy and consistency of field data collection.

From 2018 to 2024, participants underwent annual cognitive screening during community health examinations. Data were collected via in‐person interviews at community centres, linkage with health administration databases and telephone follow‐ups for non‐responders. As of August 2024, the HMACS cohort had enrolled 11 652 individuals, completed 3185 follow‐ups and recorded 538 deaths.

### Dementia ascertainment

Dementia was diagnosed by a consensus panel of two neurologists and two neuropsychologists based on the Diagnostic and Statistical Manual of Mental Disorders, Fourth Edition criteria. A diagnosis required acquired cognitive or neuropsychiatric symptoms impairing daily life, excluding delirium or major psychiatric illness, plus objective impairment in ≥ 2 of 5 domains. HMACS employed a comprehensive neuropsychological battery including the Chinese versions of the Mini‐Mental State Examination (MMSE) and Montreal cognitive assessment‐basic (global cognition), auditory verbal learning test (episodic memory), trail making test (executive function), digit span (attention), Boston naming and verbal fluency test (language) and clock drawing test (visuospatial function).^3^, ^35^, ^36^ Daily functioning was assessed using the activity of daily living scale (ADL), covering both basic and instrumental ADLs.

### Risk‐associated variables selection

From 548 variables collected via standardised case report forms, we excluded those with free‐text entries (e.g., medication names), duplicates or > 40% missing values. Remaining variables underwent Spearman's correlation analysis (*ρ* ≥ 0.7), and missing values were imputed using a random forest (RF) algorithm. To reduce dimensionality and identify key predictors, Least Absolute Shrinkage and Selection Operator (LASSO) regression was applied within a binomial logistic regression (LR) framework. The penalty parameter was first evaluated using 10‐fold cross‐validation, and a fixed *λ* value of 0.005 was then applied to enhance model sparsity. Variables with non‐zero coefficients were retained, yielding 38 key risk‐associated variables (see Supporting Information S1: table S1 and figure S1), encompassing: (1) sociodemographics: age, education, living arrangement, medical insurance, income satisfaction, familial emotional support, living siblings and number of close friends; (2) physical measurements: height, weight, waistline, right‐hand grip strength and heart rate; (3) medical history: hypertension, anaemia, head trauma, facial infection, thyroid dysfunction, cervical spondylosis, depression symptoms, Parkinson's disease, family history of diabetes and family history of stroke; (4) behaviour and lifestyle: fish consumption, fruit consumption, eating behaviours, cognitive activity and nighttime awakenings; (5) peripheral organ function assessment: hearing acuity, sense of smell, vision acuity, walking pace, gait stability, defecation habit and defecation duration and (6) subjective cognitive function: memory concerns, having worse memory than peers and seeing a doctor due to memory concerns. All 38 risk‐associated variables were obtained from routine self‐reported questionnaires, requiring no laboratory biomarkers or cognitive testing.

### Statistical analysis

Continuous variables are presented as means and standard deviations (SDs), and categorical variables as frequencies and percentages. Baseline differences between participants who developed dementia and those who remained dementia‐free were examined using independent‐samples *t* tests or Mann–Whitney *U* tests for continuous variables, and Chi‐squared tests for categorical variables, as appropriate. Variable associations were assessed using Spearman correlation, and all continuous risk‐associated variables were *Z*‐score standardised prior to machine learning. Sensitivity analyses were additionally performed by stratifying participants according to sex and age group to assess the stability of the main findings; details of the analytical procedure are provided in Supporting Information S1 and the corresponding results are presented in Supporting Information S1: figure S2. All statistical analyses were performed using R software (version 4.4.2), and a two‐sided *p* < 0.05 was considered statistically significant.

### Algorithm confirmation

#### Development of machine‐learning models

We developed the cognitive‐testing‐free dementia (Cog‐Free) risk estimation tool by evaluating eight machine learning algorithms. The best‐performing model was used to create Cog‐Free, which was then translated into a points‐based scoring system and an online calculator for easy use. This step constitutes the core of the model development of the analytical framework.

We randomly divided the HMACS cohort into a 70% training set (*n* = 2074) and a 30% internal validation set (*n* = 888). The prediction models were developed on the training set and internally validated using an independent subset from the same cohort. Using R software (version 4.4.2) and associated packages, including gbm, randomForest, xgboost, lightgbm, kknn, neuralnet, e1071, rpart, adabag and caret, we applied eight machine‐learning algorithms: LR, decision tree (DT), K‐nearest neighbors (KNN), RF, extreme gradient boosting (XGB), light gradient boosting machine (LGBM), support vector machine (SVM) and artificial neural network (NNET). Model performance was internally validated with 1000 bootstrap samples, calculating the area under the receiver operating characteristic curve (AUC), accuracy, sensitivity, specificity, negative predictive value (NPV) and positive predictive value (PPV). Evaluation encompassed receiver operating characteristic (ROC) curves for discrimination, calibration curves for agreement between estimated and observed probabilities and decision curve analysis for clinical utility across thresholds.

#### External validation of the optimal model

Finally, this optimal model was externally validated in the Chinese Longitudinal Healthy Longevity Survey (CLHLS) cohort (see eMethods, table 1 and Supporting Information S1: figure S3 for details).

**TABLE 1:** Baseline characteristics of study participants by dementia status at follow‐up

Variables	HMACS				CLHLS			
	Dementia‐free (*n* = 2670)	All‐cause dementia (*n* = 292)	Statistic	*p*‐value	Dementia‐free (*n* = 6151)	All‐cause dementia (*n* = 781)	Statistic	*p*‐value
Age (years), mean (SD)	70.52 (5.23)	72.76 (5.22)	*t* = −6.95	< 0.001	81.85 (10.62)	86.55 (10.58)	*t* = −11.70	< 0.001
Education (years), mean (SD)	7.38 (5.20)	1.57 (3.16)	*t =* 27.59	< 0.001	2.46 (3.63)	1.80 (3.12)	*t* = 5.48	< 0.001
Living arrangement, *n* (%)			*χ* ^2^ = 30.05	< 0.001			*χ* ^2^ = 7.46	0.059
Living alone	416 (15.58)	81 (27.74)			1019 (16.57)	114 (14.60)		
Living with friends or caregivers	9 (0.34)	1 (0.34)			1631 (26.52)	205 (26.25)		
Living with spouse only	1791 (67.08)	177 (60.62)			3372 (54.82)	435 (55.70)		
Generations living together	454 (17.00)	33 (11.30)			129 (2.10)	27 (3.46)		
Medical insurance, *n* (%)			*χ* ^2^ = 134.69	< 0.001				
No insurance	313 (11.72)	86 (29.45)			67 (1.09)	11 (1.41)		
Urban and rural residents' basic medical insurance	1128 (42.25)	164 (56.16)			4434 (72.09)	527 (67.48)	*χ* ^2^ = 7.01	0.008
Employees' basic medical insurance	1217 (45.58)	42 (14.38)			‐	‐		
Commercial medical insurance	12 (0.45)	0 (0.00)			‐	‐		
Income satisfaction, yes, *n* (%)	1719 (64.38)	176 (60.27)	*χ* ^2^ = 1.75	0.185	4885 (79.42)	546 (69.91)	*χ* ^2^ = 36.37	< 0.001
Familial emotional support, yes, *n* (%)	2490 (93.26)	256 (87.67)	*χ* ^2^ = 11.34	0.001	6035 (98.11)	743 (95.13)	*χ* ^2^ = 26.97	< 0.001
Living siblings, yes, *n* (%)	1285 (48.13)	123 (42.12)	χ^2^ = 3.57	0.059	3695 (60.07)	299 (38.28)	*χ* ^2^ = 133.82	< 0.001
Number of close friends, *n* (%)			*χ* ^2^ = 93.04	< 0.001				
None	602 (22.55)	128 (43.84)						
1–2	466 (17.45)	69 (23.63)						
3–5	815 (30.52)	62 (21.23)						
6 or more	787 (29.48)	33 (11.30)						
Height (cm), mean (SD)	159.61 (8.16)	153.08 (8.41)	*t* = 12.62	< 0.001	158.01 (9.37)	155.57 (10.01)	*t* = 6.46	< 0.001
Weight (kg), mean (SD)	60.75 (10.30)	54.60 (9.67)	*t* = 10.24	< 0.001	52.18 (11.11)	50.09 (10.28)	*t* = 5.30	< 0.001
Waistline (cm), mean (SD)	87.16 (9.56)	89.02 (8.90)	*t* = −3.38	0.001				
Right‐hand grip strength (kg), mean (SD)	21.97 (8.64)	14.59 (6.37)	*t* = 18.07	< 0.001				
Heart rate, mean (SD)	73.16 (9.37)	72.85 (10.30)	*t* = 0.49	0.626	73.88 (9.24)	73.02 (7.88)	*t* = 2.82	0.005
Hypertension, yes, *n* (%)	1385 (51.87)	168 (57.53)	*χ* ^2^ = 3.16	0.075	1334 (21.69)	116 (14.85)	*χ* ^2^ = 19.16	< 0.001
Anaemia, yes, *n* (%)	190 (7.12)	28 (9.59)	*χ* ^2^ = 2.01	0.156				
Head trauma, yes, *n* (%)	208 (7.79)	14 (4.79)	*χ* ^2^ = 2.99	0.084				
Facial infection, yes, *n* (%)	240 (8.99)	28 (9.59)	*χ* ^2^ = 0.05	0.816				
Thyroid dysfunction, yes, *n* (%)	157 (5.88)	6 (2.05)	*χ* ^2^ = 6.69	0.010				
Cervical spondylosis, yes, *n* (%)	907 (33.97)	92 (31.51)	*χ* ^2^ = 0.61	0.435				
Depression symptoms, yes, *n* (%)	19 (0.71)	3 (1.03)	*χ* ^2^ = 0.06	0.812				
Parkinson's disease, yes, *n* (%)	48 (1.80)	19 (6.51)	*χ* ^2^ = 24.32	< 0.001	22 (0.36)	2 (0.26)	*χ* ^2^ = 0.02	0.895
Family history of diabetes, yes, *n* (%)	370 (13.86)	35 (11.99)	*χ* ^2^ = 0.63	0.427				
Family history of stroke, yes, *n* (%)	257 (9.63)	18 (6.16)	*χ* ^2^ = 3.34	0.067				
Fish consumption, yes, *n* (%)	2455 (91.95)	231 (79.11)	*χ* ^2^ = 49.83	< 0.001	4762 (77.42)	508 (65.04)	*χ* ^2^ = 57.53	< 0.001
Fruit consumption, yes, *n* (%)	2414 (90.41)	222 (76.03)	*χ* ^2^ = 54.15	< 0.001	4748 (77.19)	512 (65.56)	*χ* ^2^ = 50.61	< 0.001
Eating behaviours, *n* (%)			*χ* ^2^ = 9.97	0.126				
Not special	2488 (93.18)	281 (96.23)						
Skipping breakfast	42 (1.57)	7 (2.40)						
Having afternoon tea	43 (1.61)	2 (0.68)						
Having midnight snacks	58 (2.17)	2 (0.68)						
Eating in the middle of the night	2 (0.07)	0 (0.00)						
Skipping lunch	12 (0.45)	0 (0.00)						
Skipping dinner	25 (0.94)	0 (0.00)						
Cognitive activity, yes, *n* (%)	1504 (56.33)	60 (20.55)	*χ* ^2^ = 133.79	< 0.001	4266 (69.35)	417 (53.39)	*χ* ^2^ = 79.83	< 0.001
Nighttime awakenings, *n* (%)			*χ* ^2^ = 7.91	0.048				
None	1389 (52.02)	127 (43.49)						
< 1 time/week	854 (31.99)	107 (36.64)						
1–2 times/week	390 (14.61)	53 (18.15)						
≥ 3 times/week	37 (1.39)	5 (1.71)						
Hearing acuity, *n* (%)			*χ* ^2^ = 0.52	0.914				
No change	2053 (76.89)	226 (77.40)						
Slight dullness	527 (19.74)	56 (19.18)						
Moderate dullness	86 (3.22)	10 (3.42)						
Significant dullness	4 (0.15)	0 (0.00)						
Sense of smell, *n* (%)			*χ* ^2^ = 0.09	0.993				
No change	2360 (88.39)	258 (88.36)						
Slight dullness	270 (10.11)	30 (10.27)						
Significant dullness	32 (1.20)	3 (1.03)						
Loss	8 (0.30)	1 (0.34)						
Vision acuity, *n* (%)			*χ* ^2^ = 45.73	< 0.001				
No change	788 (29.51)	56 (19.18)						
Slight dullness	1568 (58.73)	163 (55.82)						
Significant dullness	311 (11.65)	72 (24.66)						
Loss	3 (0.11)	1 (0.34)						
Walking pace: slower, *n* (%)	1749 (65.51)	239 (81.85)	*χ* ^2^ = 31.12	< 0.001				
Gait stability, yes, *n* (%)	2386 (89.36)	217 (74.32)	*χ* ^2^ = 54.56	< 0.001				
Defecation habit, *n* (%)			*χ* ^2^ = 8.24	0.041				
Morning	2005 (75.09)	197 (67.47)						
Afternoon	46 (1.72)	7 (2.40)						
Night	49 (1.84)	8 (2.74)						
Irregular	570 (21.35)	80 (27.40)						
Defecation duration (min), mean (SD)	8.67 (6.52)	9.98 (9.17)	*t* = −2.39	0.002				
Memory concerns, yes, *n* (%)	1066 (39.93)	145 (49.66)	*χ* ^2^ = 9.92	0.002				
Having worse memory than peers, yes, *n* (%)	682 (25.54)	130 (44.52)	*χ* ^2^ = 46.69	< 0.001				
Seeing a doctor due to memory concerns, yes, *n* (%)	74 (2.77)	7 (2.40)	*χ* ^2^ = 0.03	0.855				

*Note*: Data are presented as mean (SD) or *n* (%).

Abbreviations: CLHLS, Chinese Longitudinal Healthy Longevity Survey; HMACS, Hubei Memory and Aging Cohort Study; SD, standard deviation.

#### Model interpretability and visualisation via SHAP

Model interpretability was achieved using SHapley Additive exPlanations (SHAP), which quantified variable contributions and visualised feature importance and directional effects via bar plots and beeswarm plots.

### Comparison with existing risk estimation tools

We compared the proposed model with three established dementia risk estimation tools, CogDrisk, ANU‐ADRI and Modified‐LIBRA (see Supporting Information S1: table S2), using the imputed HMACS test set. All three tools were designed for adults aged 65 and older: CogDrisk assesses dementia risk in individuals ≥ 65^25^; ANU‐ADRI was developed for older populations^21^ and Modified‐LIBRA, though initially studied in former NFL players aged ≥ 50, applies to those over 65.^23^ The CAIDE risk score was excluded as it targets middle‐aged adults (40–60 years).^37^ Differences in AUC were assessed for statistical significance using DeLong's test. This comparative analysis was designed not merely as a validation step but as part of the methodological evaluation of the proposed analytical framework.

## RESULTS

### Participant characteristics

This study included 2962 non‐dementia older adults (cognitively normal or with MCI) from HMACS at baseline (54.99% female; mean age 71.64 [5.27] years). Significant baseline differences were observed between the included and excluded (*n* = 8690) cohorts (Supplementary table S3). Compared with the excluded cohort, the included cohort was older, had less formal education and lived alone more frequently. Although they reported more memory concerns and a higher burden of certain health conditions such as hypertension, they exhibited healthier lifestyle patterns, including higher consumption of fish and fruit.

The analysis cohort was randomly split into a training set (*n* = 2074) and an internal validation set (*n* = 888). Over a mean (SD) follow‐up of 2.18 (1.25) years, 292 all‐cause dementia cases (9.86%) were identified. As summarised in table 1, participants who developed dementia were typically older, less educated, had weaker right‐hand grip strength, were shorter and were less cognitively active than those who remained dementia‐free. They also reported subjective memory complaints more frequently, such as having worse memory than peers (44.52% vs. 25.54%) and general memory concerns (49.66% vs. 39.93%).

### Algorithm confirmation

The eight machine‐learning models showed varying AUCs in the training set: LR: 0.91 (95% confidence interval [CI] 0.89–0.92), KNN: 1.00 (95% CI 1.00–1.00), DT: 0.50 (95% CI 0.50–0.50), RF: 1.00 (95% CI 1.00–1.00), XGB: 0.94 (95% CI 0.93–0.95), LGBM: 0.99 (95% CI 0.99–1.00), SVM: 0.94 (95% CI 0.93–0.96) and NNET: 0.91 (95% CI 0.89–0.93) (Supporting Information S1: table S4). In the test set, all models except KNN and DT maintained reasonable performance, with AUCs of 0.86 (95% CI 0.82–0.89), 0.59 (95% CI 0.55–0.64), 0.50 (95% CI 0.50–0.50), 0.85 (95% CI 0.81–0.88), 0.85 (95% CI 0.81–0.88), 0.84 (95% CI 0.80–0.88), 0.84 (95% CI 0.80–0.87) and 0.80 (95% CI 0.75–0.84), respectively (table 2). KNN showed significant overfitting (training AUC = 1.00 vs. test AUC = 0.59), whereas DT performed no better than random guessing (AUC = 0.50). The remaining six models (LR, RF, XGB, LGBM, SVM and NNET) showed no evident overfitting, with sensitivity from 0.39 to 0.78 and specificity from 0.77 to 0.93, high NPV (0.93–0.97) and generally low PPV (0.27–0.37).

**TABLE 2:** Model performance for predicting incident dementia in testing datasets

Models	AUC (95% CI)	Accuracy (95% CI)	Sensitivity (95% CI)	Specificity (95% CI)	PPV (95% CI)	NPV (95% CI)
HMACS, 38 key features after LASSO selection						
LR	0.86 (0.82–0.89)	0.81 (0.78–0.83)	0.78 (0.68–0.85)	0.81 (0.78–0.84)	0.32 (0.26–0.38)	0.97 (0.95–0.98)
KNN	0.59 (0.55–0.64)	0.85 (0.83–0.87)	0.26 (0.18–0.35)	0.92 (0.90–0.94)	0.26 (0.18–0.36)	0.92 (0.90–0.93)
DT	0.50 (0.50–0.50)	0.90 (0.88–0.92)	0.00 (0.00–0.04)	1.00 (1.00–1.00)	‐	0.90 (0.88–0.92)
RF	0.85 (0.81–0.88)	0.77 (0.74–0.80)	0.77 (0.67–0.84)	0.77 (0.74–0.80)	0.27 (0.22–0.33)	0.97 (0.95–0.98)
XGB	0.85 (0.81–0.88)	0.79 (0.76–0.81)	0.73 (0.63–0.81)	0.79 (0.76–0.82)	0.28 (0.23–0.34)	0.96 (0.95–0.98)
LGBM	0.84 (0.80–0.88)	0.81 (0.78–0.83)	0.66 (0.55–0.75)	0.83 (0.80–0.85)	0.30 (0.24–0.36)	0.96 (0.94–0.97)
SVM	0.84 (0.80–0.87)	0.77 (0.74–0.80)	0.76 (0.66–0.83)	0.77 (0.74–0.80)	0.27 (0.22–0.33)	0.97 (0.95–0.98)
NNET	0.80 (0.75–0.84)	0.87 (0.85–0.89)	0.39 (0.30–0.49)	0.93 (0.90–0.94)	0.37 (0.28–0.47)	0.93 (0.91–0.95)

Abbreviations: AUC, area under the receiver operating characteristic curve; CI, confidence interval; DT, decision tree; HMACS, Hubei Memory and Aging Cohort Study; KNN, K‐nearest neighbors; LASSO, Least Absolute Shrinkage and Selection Operator; LGBM, light gradient boosting machine; LR, logistic regression; NNET, artificial neural network; NPV, negative predictive value; PPV, positive predictive value; RF, random forest; SVM, support vector machine; XGB, extreme gradient boosting.

**FIGURE 3:**
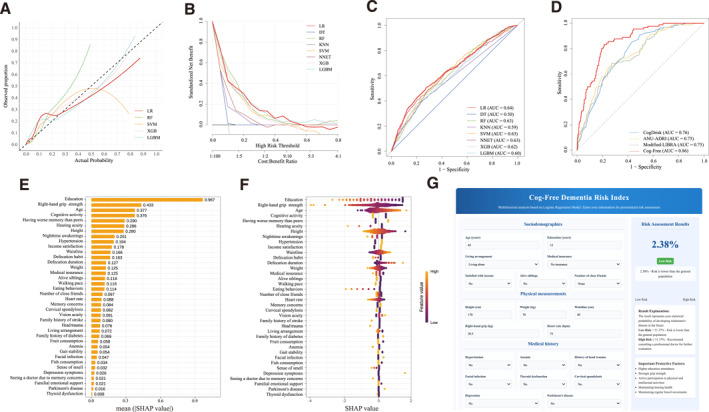
Comprehensive evaluation and visualisation of machine‐learning models for dementia risk estimation. (A) Calibration curves of machine‐learning models for estimating dementia in the elderly. (B) Decision curves of machine‐learning models for estimating dementia. (C) ROC curves of the eight simplified estimation models evaluated on the testing set. The HMACS cohort served as the training set, whereas the Chinese Longitudinal Healthy Longevity Survey Cohort was employed as the test set. (D) ROC curves comparing the LR model with the CogDrisk, ANU‐ADRI and Modified‐LIBRA models on the testing set (30% of the HMACS cohort). (E) Bar chart of feature importance for the LR model. (F) Swarm plot of feature importance for the LR model. (G) Interface of the Cog‐Free web application. ANU‐ADRI, Australian National University Alzheimer’s Disease Risk Index; AUC, area under the receiver operating characteristic curve; CogDrisk, Cognitive Health and Dementia Risk Index; Cog‐Free, cognition‐free model; DT, decision tree; HMACS, Hubei Memory and Aging Cohort Study; KNN, K‐nearest neighbors; LGBM, light gradient boosting machine; LR, logistic regression; Modified‐LIBRA, modified Lifestyle for Brain Health index; NNET, artificial neural network; RF, random forest; ROC, receiver operating characteristic; SHAP, SHapley Additive exPlanations; SVM, support vector machine; XGB, extreme gradient boosting.

Calibration curves (figure 3A) indicated that LR and LGBM had the best alignment between identified and observed risks. Decision curve analysis (figure 3B) further supported LR as having the highest net benefit across most risk thresholds, suggesting superior clinical utility, with LGBM also performing well in certain ranges. Overall, LR excelled in both estimative accuracy and clinical applicability. External validation results (figure 3C) were consistent with these findings (see Supporting Information S1: tables S5 and S6 for details).

### Tool comparison with existing risk estimation tools

The Cog‐Free dementia risk tool was compared with three established dementia risk estimation tools: CogDrisk, ANU‐ADRI and Modified‐LIBRA.^21^, ^23^, ^25^ As shown in figure 3D and Supporting Information S1: table S7, Cog‐Free achieved an AUC of 0.86 (95% CI 0.82–0.89), significantly outperforming CogDrisk (0.76 [95% CI 0.72–0.80]), ANU‐ADRI (0.75 [95% CI 0.70–0.80]) and Modified‐LIBRA (0.75 [95% CI 0.70–0.79]). It also demonstrated superior sensitivity (0.78 [95% CI 0.68–0.85]), specificity (0.81 [95% CI 0.78–0.84]) and precision (0.81 [95% CI 0.78–0.83]). DeLong's test confirmed that these AUC differences were statistically significant (all *p* < 0.001), supporting Cog‐Free's superior discriminative ability.

### Feature importance ranking

SHAP analysis was used to interpret both global and individual feature importance in the Cog‐Free tool (figure 3E, F). Globally, the most influential risk‐associated variables included years of education, right‐hand grip strength, age, cognitive activity, self‐reported worse memory than peers, hearing acuity, height and nighttime awakenings. At the individual level, factors such as hypertension and income satisfaction exerted consistent though modest effects, whereas eating behaviours, depressive symptoms and heart rate showed stronger impacts within specific subgroups.

The beeswarm plot illustrates the distribution and direction of each feature's influence across participants, with colour indicating variable value. SHAP values on the *x*‐axis reflect the magnitude and direction of effect on dementia risk, with higher values indicating increased dementia risk. For example, lower grip strength (yellow) was associated with a higher risk (rightward shift), whereas stronger grip (purple) corresponded to reduced risk. Similar patterns were observed for other risk‐associated variables: reduced education, advanced age, cognitive inactivity, shorter height, hypertension, income satisfaction, larger waistline, specific defecation habits, underweight status, lack of medical insurance, social isolation, slower gait, low heart rate, memory concerns, impaired vision, family history of diabetes, poor diet, gait instability, facial infection, depressive symptoms, medical consultation for memory issues and Parkinson's disease. These were all linked to increased dementia risk. In contrast, poorer hearing acuity, more nighttime awakenings, cervical spondylosis, family history of stroke, head trauma, anaemia, diminished sense of smell, thyroid dysfunction and skipping dinner were associated with a lower risk.

### Web application

To support practical implementation, we deployed a bilingual (Chinese/English) web‐based calculator for Cog‐Free (figure 3G), available at: https://cheche‐cog.github.io/cogfree/. The tool enables users to enter 38 risk‐associated variables and obtain a personalised dementia risk estimate within 5 min, allowing seamless integration with community health record systems.^38^

## DISCUSSION

### Main findings

This study proposes a data‐driven analytical framework for cognitive‐testing‐free dementia risk estimation using routine health examination data. By integrating LASSO‐based feature selection, multi‐algorithm benchmarking and SHAP interpretability analysis, the method provides a transparent and reproducible approach to risk modelling that can be adapted to other diseases and populations.

A common limitation of existing indices (e.g., ANU‐ADRI and CogDrisk) lies in their reliance on expert‐defined variables and pre‐assigned weights. By comparison, the Cog‐Free framework uses data to drive variable selection, thereby revealing novel risk‐associated variables such as right‐hand grip strength and defecation timing. This analytical approach improves generalisability and demonstrates the potential of routinely collected, non‐cognitive data for assessing dementia risk.

Using data from the HMACS, we applied this framework to develop the Cog‐Free model and externally validated it in the CLHLS cohort. Cog‐Free was built using easily accessible community health examination data and demonstrated strong discriminative performance, with an AUC of 0.86 (95% CI 0.82–0.89), specificity of 0.81 (95% CI 0.78–0.84), and overall accuracy of 0.81 (95% CI 0.78–0.83). It has been implemented as a web‐based calculator that integrates seamlessly into community hospital health platforms, enabling dynamic dementia risk assessment within existing electronic health record systems. This study suggests that community screening could use the Cog‐Free tool to assess individual dementia risk based on high‐risk factors such as advanced age, hypertension and insomnia.^14^, ^15^, ^20^ High‐risk individuals could then be prioritised for further cognitive assessment using tools such as the MMSE. Although the present study did not directly evaluate implementation outcomes, Cog‐Free has the potential to enhance accessibility and efficiency of initial dementia risk stratification, particularly in settings where professional cognitive assessment resources are limited. Nevertheless, the relatively large number of variables required by the full model may limit feasibility in certain resource‐poor settings. Future work will focus on variable reduction and the development of simplified versions of the tool to improve ease of implementation while maintaining acceptable performance. As such, it may be suitable for application in low‐ and middle‐income countries and regions^39^, ^40^ and may help address current gaps in early dementia screening coverage.

All risk‐associated variables incorporated in Cog‐Free are supported by established theoretical evidence. Beyond the 14 major risk factors highlighted by the Lancet standing commission, such as lower educational attainment, hypertension, decreased hearing acuity and depression symptoms,^1^ this study identified several previously underemphasized variables closely related to cognitive status. Self‐perceived memory concerns and reports of worse memory than peers suggest that subjective perception may serve as a sensitive early warning signal, valuable for targeting early interventions. Participants without medical insurance had a significantly higher dementia risk than those covered by employee insurance, reflecting socioeconomic disparities and underscoring the roles of healthcare access and health literacy. This finding points to potential policy‐level intervention opportunities.^41^ Bowel habits (morning bowel movement and longer defecation duration) may signal altered gut motility and microbial dysbiosis, with potential links to dementia risk via the gut–brain axis. Such changes can diminish beneficial metabolites such as short‐chain fatty acids, impair gut barrier function and drive systemic inflammation, ultimately fostering neuroinflammatory processes associated with dementia.^42^ Social variables such as living arrangements and familial emotional support are particularly relevant in the Chinese context, where family structure and social support play crucial roles in cognitive ageing.^43^ Epidemiological studies have established an association between cognitive inactivity and an increased risk of dementia.^35^, ^44^ Incorporating these factors enhances the tool's cultural and contextual relevance.

SHAP analysis further revealed two distinct types of risk‐associated variables, informing intervention strategies at both population and individual levels. First, population‐strong/individual‐weak variables (e.g., hypertension and income satisfaction) showed high overall contribution but limited variability in individual impact. This supports their primary utility in population‐level risk stratification. For example, hypertension, a known cerebrovascular risk factor, elevates dementia incidence at the population level through long‐term cerebral small vessel damage and hypoperfusion.^45^ Thus, public health measures such as blood pressure control programs are emphasised, though individual risk assessment should still incorporate multiple factors. Lower income satisfaction may predict poorer cognitive health because it reflects persistent subjective financial strain beyond income alone. This strain can elevate chronic stress, limit access to healthcare and health‐promoting resources, impair diet and disease management and reduce social participation, all of which compromise cognitive deterioration.^46^, ^47^, ^48^ Second, population‐weak/individual‐strong variables (e.g., eating behaviours, depressive symptoms and heart rate) had lower overall importance but exhibited wide variability across individuals. These may serve as critical targets for intervention for specific high‐risk subgroups. For instance, although depression may contribute less at the population level than age or education, chronic depression can activate the hypothalamic–pituitary–adrenal axis and raise cortisol levels, directly damaging the hippocampus and accelerating cognitive decline.^49^ Therefore, individuals presenting depression alongside other risk factors (e.g., weaker right‐hand grip strength) warrant targeted clinical attention.

In this study, several established dementia risk factors from previous epidemiological studies, including decreased hearing acuity, head trauma,^50^ nighttime awakenings^51^, ^52^ and anaemia,^53^ were negatively associated with dementia risk in our model. These inverse associations likely reflect cohort‐ and measurement‐specific effects and should not be interpreted as evidence that these factors are protective against dementia. Other factors frequently linked to cognitive impairment in prior work, such as cervical spondylosis, family history of stroke, and reduced sense of smell^54^, ^55^ also showed no consistent effects here. Several factors may explain these discrepancies observed between the present results and prior epidemiological findings. This longitudinal study followed a community‐based cohort of initially non‐demented older adults, and the generally mild severity of these conditions in the sample may have led to biased risk estimates. As HMACS is a large cluster‐sampled community cohort, corresponding preventive measures for identified risk factors were provided to participants during cognitive screening.^35^ Through peer communication and earlier awareness of these risk factors, older adults might seek interventions sooner. Previous studies report that those with weaker grip strength show greater willingness to engage in multi‐sensory rehabilitation training,^53^ and hearing‐impaired older adults may reduce dementia risk through hearing‐aid use.^56^ Additionally, the Cog‐Free model simultaneously adjusted for numerous social, lifestyle and biological factors. Interactions between certain variables, such as high education level, active social engagement and cognitive activities, may have partially attenuated the true risk estimates of these factors. Finally, as most information was self‐reported or measured only at baseline, recall bias and measurement error may have led to an underestimation of the association between mild symptoms and disease status.

This study has several strengths. Based on the HMACS, which includes urban and rural populations from less developed central China, it reflects health characteristics of older adults in low‐ and middle‐income settings. The required variables can be obtained using basic medical equipment available in primary care. The tool also circumvents the psychological resistance often associated with traditional cognitive screening scales, potentially improving compliance and population coverage. Compared to classic cognitive‐testing‐free tools such as CogDrisk,^25^ which was derived from meta‐analyses and incorporated only predefined risk factors, our data‐driven approach selected risk‐associated variables from multidimensional health data, broadening variable coverage to capture more aspects of overall health and their influence on cognitive function. Notably, we identified several previously under‐recognised risk‐associated variables of dementia risk, including right‐hand grip strength, self‐perceived worse memory than peers, income satisfaction and defecation timing. In direct performance comparisons, Cog‐Free also demonstrated a higher AUC and stronger overall performance. Finally, the tool has been developed into an easy‐to‐use web application that requires only brief training for community healthcare workers, greatly reducing implementation barriers.^32^

### Limitations

However, this study has limitations. The 2‐year follow‐up period, although effective for identifying short‐term conversion risk, may underestimate the estimative power for slowly progressive cognitive disorders, because typical Alzheimer’s disease pathology accumulates over 10–20 years.^57^ Future work should extend the follow‐up to verify the tool's stability in estimating long‐term risk. Moreover, our dataset carries potential biases: many risk‐associated variables were self‐reported, introducing recall and reporting errors; all participants were aged ≥ 65 years, limiting generalisability to younger populations; and missing data were handled using random forest imputation. If data are not missing at random, for example, if high‐risk individuals avoid grip strength tests due to mobility issues, imputed values may systematically underestimate risk. In addition, outcome definitions differed between the CLHLS and HMACS cohorts. This variation mainly influenced data distribution and may have caused minor performance fluctuations, but it does not invalidate the external validation results. Validation using datasets with consistent outcome definitions would yield more robust findings. The absence of several key variables in the external validation dataset, such as right‐hand grip strength and gait stability, may have influenced risk estimation and potentially led to an underestimation of dementia risk. Sensitivity analyses stratified by sex and age group were performed because age and sex are key demographic factors associated with heterogeneity in dementia risk and may influence model performance in older populations. Analyses excluding participants who were close to a dementia diagnosis at baseline were not conducted because the follow‐up period was relatively short, and applying this restriction would have substantially reduced the analytical sample size. Future studies with longer follow‐up are needed to examine this issue further. Future studies should seek external validation cohorts with greater availability of variables to further assess the robustness and generalisability of the Cog‐Free model. Standardised variable collection is needed to enhance the tool's generalisability. Finally, some established risk factors (e.g., hearing loss, head trauma, nighttime awakenings and anaemia) showed negative associations in our model, likely due to mild symptom severity, confounding by social and lifestyle variables and potential recall or measurement bias. Future studies with larger, well‐stratified cohorts and repeated objective assessments are needed to clarify these relationships.

### Implications

This study proposes a data‐driven analytical method for cognitive‐testing‐free dementia risk estimation using routinely collected health examination data. The method relies solely on common clinical and lifestyle variables, reducing the need for professional cognitive assessments and includes a web‐based calculator to enhance accessibility and practical use. It offers a reproducible approach that can be extended to risk estimation in other disease domains.

## AUTHOR CONTRIBUTIONS

Jiwen Che, Na Liu and Guirong Cheng: concept and design. Jiwen Che and Na Liu: analysis, interpretation of data and statistical analysis. All authors: drafting of the manuscript, critical revision of the manuscript for important intellectual content and guarantor.

## FUNDING

The funding for this study was provided by Brain Science and Brain‐like Intelligence Technology‐National Science and Technology Major Project (Grant Number: 2022ZD0211600), and the National Natural Science Foundation of China (Grant Numbers: 72174159 and 82171491).

## ETHICS STATEMENT

The study was approved by the Medical Ethics Committee of Wuhan University of Science and Technology (Approval No.: 201845). All participants provided written informed consent.

## Supporting information

Supporting Information S1
