# Supplementary material for: Development and validation of a Cog‐Free risk predicting tool for dementia in a community setting
Source: Gen Psychiatr. 2026 Jun 1;39(3):e70028. doi: 10.1002/gps3.70028 (PMC13240457; doi:10.1002/gps3.70028)
Supplement: Supplementary file 1 — Supporting Information S1 [file GPS3-39-e70028-s001.docx]

**eMethods**

**External validation**

We externally validated the model in 7,813 adults aged 65+ from the Chinese Longitudinal Healthy Longevity Survey (CLHLS), a nationwide study (2008–2011) across 23 Chinese regions (37, 38). Dementia was defined as a Chinese MMSE score <18 plus impairment in ≥1 basic or instrumental activity of daily living (39). Over a mean follow-up of 3.08 (0.21) years, 267 all-cause dementia cases (4.7%) were identified. The study was approved by Peking University (IRB00001052-13074).

To address low dementia prevalence (4.7%) in the CLHLS, we applied the Synthetic Minority Over-sampling Technique-Edited Nearest Neighbors (SMOTE-ENN) hybrid sampling algorithm to balance the validation set, adjusting the positive rate to 11.2% for better comparability with the Hubei Memory and Aging Cohort Study (HMACS) training set.

As CLHLS lacked some risk-associated variables available in HMACS, we selected 15 shared variables for validation: age, education, living arrangement, medical insurance, income satisfaction, alive siblings, height, weight, heart rate, hypertension, Parkinson’s disease, fish consumption, fruit consumption, cognitive activity, and familial emotional support. Models trained on HMACS were validated on the processed CLHLS set. Performance was assessed using the receiver operating characteristic curve, accuracy, sensitivity, specificity, positive predictive value, and negative predictive value, with a focus on whether logistic regression remained the optimal algorithm.

As CLHLS lacked some risk-associated variables available in HMACS, 15 shared features were selected for external validation: age, educational level, living arrangement, medical insurance, income satisfaction, alive siblings, height, weight, heart rate, hypertension, Parkinson’s disease, fish consumption, fruit consumption, cognitive activity, and familial emotional support. Models were retrained using HMACS and validated on the processed CLHLS set. Performance was evaluated using AUC, accuracy, sensitivity, specificity, PPV, and NPV, with emphasis on whether logistic regression (LR) remained the optimal algorithm.

**Multivariable logistic regression and risk score calculation**

Supplementary Table 8 summarizes the results of the multivariable logistic regression analysis examining associations between baseline predicting features and incident dementia. Older age was significantly associated with increased dementia risk (β = 0.088, P < 0.001), whereas higher educational attainment was protective (β = -0.204, P < 0.001). Participants with greater income satisfaction (β = 0.388, P = 0.044) and hypertension (β = 0.390, P = 0.038) had higher dementia risk. In contrast, higher right-hand grip strength (β = -0.068, P < 0.001), more frequent cognitive activity (β = -0.754, P = 0.001), better hearing acuity (β = –0.661, P = 0.002), and regular defecation habits (β = -0.148, P = 0.036) were associated with lower risk of dementia. In addition, longer defecation duration (β = 0.027, P = 0.007) and perceiving one’s memory as worse than peers (β = 0.731, P < 0.001) were related to increased risk. Additional variables were retained in the model based on prior evidence and clinical relevance to ensure comprehensive risk estimation. These findings were consistent with the SHAP analysis, which confirmed the direction and relative importance of the key risk-associated variables.

The coefficients (Estimates) presented in Supplementary Table 8 also served as the basis for calculating the individualized dementia risk scores. For each participant, the total risk score was derived by summing the products of each variable’s value and its corresponding coefficient, plus the intercept term, and then transformed into a probability value using the Sigmoid function.

The optimal cutoff value was identified via ROC curve analysis, specifically by maximizing the Youden’s index (sensitivity + specificity − 1) for optimal overall performance and balancing sensitivity with specificity to equate the risks of misdiagnosis and missed diagnosis. This analysis yielded an optimal cutoff of 0.055, corresponding to a risk threshold score of 51.37%.

Participants were classified into risk groups using this threshold: those with a risk score (risk score) ≥ 51.37% were defined as the high-risk group, while those with a risk score < 51.37% were classified as the low-risk group.

**Sensitivity analysis**Sensitivity analyses were conducted to evaluate the stability of the main findings. Participants were stratified by sex, and were also divided into younger and older age groups according to the mean age of the study population. The full modeling procedure used in the primary analysis was then repeated within each subgroup.

**eResults**

**External validation**

Across the eight machine learning models developed using HMACS, AUC values in the training set were as follows: LR: 0.863 (0.844, 0.883), KNN: 1.000 (0.999, 1.000), DT: 0.500 (0.500, 0.500), RF: 1.000 (0.999, 1.000), XGB: 0.923 (0.908, 0.937), LGBM: 0.953 (0.944, 0.961), SVM: 0.964 (0.952, 0.974), and NNET: 0.881 (0.856, 0.901) (Supplementary Table 6). In the test set using the CLHLS, all models showed the following performances: LR: AUC = 0.644 (0.622, 0.666), KNN: AUC = 0.585 (0.565, 0.604), DT: AUC = 0.500 (0.500, 0.500), RF: AUC = 0.626 (0.605, 0.646), XGB: AUC = 0.617 (0.597, 0.638), LGBM: AUC = 0.600 (0.579, 0.622), SVM: AUC = 0.628 (0.608, 0.647), NNET: AUC = 0.631 (0.609, 0.651) (Supplementary Table 7).

DT performed no better than random guessing (AUC = 0.500). No evident overfitting was observed in LR, KNN, RF, XGB, LGBM, SVM, or NNET. Across these seven stable models, sensitivity ranged from 0.279 to 0.859, specificity from 0.264 to 0.838, PPV from 0.127 to 0.135, and NPV from 0.921 to 0.941.

In the simplified model retrained using 15 risk-associated variables shared by both HMACS and CLHLS, despite a reduction in the number of risk-associated variables, the top seven important features remained largely consistent, with only two excluded (right-hand grip strength and memory concerns). The LR model demonstrated consistently superior performance compared to the other seven algorithms across both internal and external validation sets (Supplementary Figure 2C, Supplementary Tables 5-6).

**Sensitivity analysis**In the test set, among males (Supplementary Figure 2B), XGBoost achieved the highest performance (AUC = 0.82), followed by logistic regression (AUC = 0.81), LightGBM (AUC = 0.81), random forest (AUC = 0.79),SVM (AUC = 0.76), and neural network (AUC = 0.76). KNN showed lower discrimination (AUC = 0.53), and decision tree had the lowest performance (AUC = 0.50). Among females (Supplementary Figure 2D), logistic regression showed the highest performance (AUC = 0.87), followed by XGBoost (AUC = 0.87),SVM (AUC = 0.85), LightGBM (AUC = 0.85), random forest (AUC = 0.84), and neural network (AUC = 0.84). KNN yielded an AUC of 0.70, whereas decision tree again showed the lowest performance (AUC = 0.50).

Among the younger age group (Supplementary Figure 2F), logistic regression achieved the highest performance (AUC = 0.90), followed by XGBoost (AUC = 0.87), random forest (AUC = 0.87), LightGBM (AUC = 0.87), neural network (AUC = 0.86), andSVM (AUC = 0.86). KNN showed relatively low discrimination (AUC = 0.57), and decision tree had the lowest performance (AUC = 0.50). In the older age group (Supplementary Figure 2H), XGBoost achieved the highest performance (AUC = 0.86), followed by LightGBM (AUC = 0.86), random forest (AUC = 0.84), logistic regression (AUC = 0.82), and neural network (AUC = 0.82).SVM showed moderate discrimination (AUC = 0.80), whereas KNN (AUC = 0.63) and decision tree (AUC = 0.50) performed less well.

SUPPLEMENTARY TABLE 1 Definitions and assessments of variables in the Hubei Memory and Aging Cohort Study.

| **Predicting variables** | **Definitions and assessments** | **Measurement** level |
| --- | --- | --- |
| **Sociodemographics** | | |
| Age | Based on the participant’s Chinese resident identity card. | Scale |
| Education | Self-reported school education years. | Scale |
| Living arrangement | Self-reported living arrangement was classified as: 0 = living alone, 1 = living with friends or caregivers, 2 = living with spouse only, and 3 = generations living together. | Ordinal |
| Medical insurance | Self-reported medical insurance was classified as follows: 0 = no insurance, 1 = urban and rural residents’ basic medical insurance, 2 = employees’ basic medical insurance, and 3 = commercial medical insurance. | Ordinal |
| Income satisfaction | The self-reported status of being satisfied with current personal income was coded as follows: 0 = not satisfied with income, 1 = satisfied with income. | Ordinal |
| Familial emotional support | Self-reported family emotional support was coded as 0 = no, 1 = yes. | Ordinal |
| Alive siblings | Self-reported information regarding having living siblings was classified as: 0 = none, 1 = one or more. | Ordinal |
| Number of close friends | The self-reported number of close friends they could talk to was categorized as follows: 0 = none, 1 = one to two, 2 = three to five, and 3 = six or more. | Ordinal |
| **Physical measurements** | | |
| Height | Height was measured in centimeters using a stadiometer with the participant standing upright, barefoot, and heels, buttocks, and shoulders touching the vertical board. Measurements were taken in duplicate, and the average was used for analysis. | Scale |
| Weight | Weight was measured in kilograms (kg) using a digital scale with the participant wearing light clothing and no footwear. Measurements were taken in duplicate, and the average was recorded to the nearest 0.1 kg. | Scale |
| Waistline | Waistline was measured in triplicate to the nearest millimeter and averaged for analysis. | Scale |
| Right-hand grip strength | Right-hand grip was measured in kilograms using a handheld dynamometer. Participants were instructed to stand with their arms at their sides, elbows flexed at 90 degrees, and grip the dynamometer as tightly as possible. Three measurements were taken for each hand, and the highest value of the right hand was used for analysis. | Scale |
| Heart rate | Heart rate was measured in beats per minute using the heart rate function displayed on a sphygmomanometer (blood pressure monitor) device. Participants rested in a seated position for five minutes before measurement, and the average of three consecutive readings was recorded. | Scale |
| SUPPLEMENTARY TABLE 1 (continued) | | |
| **Predicting variables** | **Definitions and assessments** | **Measurement level** |
| **Medical history** | | |
| Hypertension | Preferred self-reported clinical diagnosis of hypertension and/or the use of any antihypertensive medication within two weeks; if not, measured the value of blood pressure to define hypertension (systolic BP ≥140 mm Hg, and/or diastolic BP ≥ 90 mm Hg) based on the national clinical practice guidelines on the management of hypertension in primary health care. Responses were classified as: 0 = no, 1 = yes. | Ordinal |
| Anemia | Self-reported clinical diagnosis of anemia was classified as follows: 0 = no, 1 = yes. | Ordinal |
| Head trauma | Self-reported traumatic brain injury was classified as: 0 = no, 1 = yes. | Ordinal |
| Facial infection | Self-reported facial infection was classified as: 0 = no, 1 = yes. | Ordinal |
| Thyroid dysfunction | Self-reported clinical diagnosis of thyroid dysfunction was classified as: 0 = no, 1 = yes. | Ordinal |
| Cervical spondylosis | Self-reported clinical diagnosis of cervical spondylosis was classified as: 0 = no, 1 = yes. | Ordinal |
| Depression symptoms | Evaluated depression symptoms using GDS-15 were classified as: 0 = 0-4 scores, 1 = 5-15 scores. | Ordinal |
| Parkinson’s disease | Self-reported clinical diagnosis of Parkinson's disease was classified as: 0 = no, 1 = yes. | Ordinal |
| Family history of diabetes | Self-reported family history of diabetes was classified as: 0 = no, 1 = yes. | Ordinal |
| Family history of stroke | Self-reported family history of stroke was classified as: 0 = no, 1 = yes. | Ordinal |
| **Behavior and lifestyle** | | |
| Fish consumption | Self-reported fish consumption habits were classified as: 0 = no, 1 = yes. | Ordinal |
| Fruit consumption | Self-reported fruit consumption was classified as: 0 = no, 1 = yes. | Ordinal |
| Eating behaviors | Self-reported eating behaviors were categorized into the following labels: 0 = not special, 1 = skipping breakfast, 2 = having afternoon tea, 3 = having midnight snacks, 4 = eating in the middle of the night, 5 = skipping lunch, 6 = skipping dinner. | Ordinal |
| Cognitive activity | Self-reported participation in cognitive activity was classified as: 0 = cognitively inactive, 1 = cognitively active. | Ordinal |
| Nighttime awakenings | Self-rated frequency of nighttime awakenings in the past month was scored as follows: 0 = none, 1 = < 1 time/week, 2 = 1–2 times/week, 3 = ≥ 3 times/week. | Ordinal |
| **Peripheral organ function assessment** | | |
| Hearing acuity | Self-reported current hearing in comparison to before was coded as 0 = no change, 1 = slight dullness, 2 = significant dullness, and 3 = loss. | Ordinal |
| Sense of smell | Self-reported current sense of smell compared to before was coded as 0 = no change, 1 = slight dullness, 2 = significant dullness, and 3 = loss. | Ordinal |
| SUPPLEMENTARY TABLE 1 (continued) | | |
| **Predicting variables** | **Definitions and assessments** | **Measurement** level |
| Vision acuity | Self-reported current vision in comparison to before was coded as 0 = no change, 1 = slight dullness, 2 = significant dullness, and 3 = loss. | Ordinal |
| Walking pace | Slower step frequency compared to a previous measurement was coded as 1 = slower, 2 = normal. | Ordinal |
| Gait stability | Measured gait instability was coded as: 0 = no, 1 = yes | Ordinal |
| Defecation habit | Defecation habits were classified into four levels: 1 = morning, 2 = afternoon, 3 = night, 4 = irregular. | Ordinal |
| Defecation duration | Self-reported duration of defecation (in minutes) per episode for the designated period was recorded. | Scale |
| **Subjective cognitive function** | | |
| Memory concerns | Self-reported concern about memory decline was coded as 0 = no, 1 = yes. | Ordinal |
| Having worse memory than peers | Self-reported comparative severity of memory decline relative to peers was coded as 0 = no, 1 = yes. | Ordinal |
| Seeing a doctor due to memory concerns | Self-reported doctor consultation due to memory concerns was coded as 0 = no, 1 = yes. | Ordinal |

SUPPLEMENTARY TABLE 2 Description of the covariates used in the dementia risk assessment tools of CogD points, ANU-ADRI points, and Modified-LIBRA^1^.

|  | **CogD points** | **ANU-ADRI points** | **Modified-LIBRA** |
| --- | --- | --- | --- |
| Age for males (years) |  |  |  |
| 60-64 | 0 | 0 | 0 |
| 65-69 | 6 | 1 | 1 |
| 70-74 | 8 | 12 | 12 |
| 75-79 | 13 | 18 | 18 |
| 80-84 | 17 | 26 | 26 |
| 85-89 | 20 | 33 | 33 |
| >90 | 22 | 38 | 38 |
| Age for females (years) |  |  |  |
| 60-64 | 0 | 0 | 0 |
| 65-69 | 4 | 5 | 5 |
| 70-74 | 7 | 14 | 14 |
| 75-79 | 11 | 21 | 21 |
| 80-84 | 15 | 29 | 29 |
| 85-89 | 19 | 35 | 35 |
| >90 | 23 | 41 | 41 |
| Education level, n (%) |  |  |  |
| Primary | 4 | 6 | 6 |
| Secondary | 2 | 3 | 3 |
| Tertiary | 0 | 0 | 0 |
| Midlife (<=65 years) obesity, n% |  |  |  |
| Under weight | 2 | - | - |
| Normal | 0 | 0 | 0 |
| Overweight | 1 | 2 | 1.6 |
| Obese | 3 | 5 | 1.6 |
| Diabetes |  |  |  |
| Yes | 2 | 3 | 1.3 |
| Depression (CESD>16) |  |  |  |
| Yes | 3 | 2 | 2.1 |
| High cholesterol (aged <60) |  |  |  |
| Yes | 3 | 3 | 1.4 |
| TBI |  |  | NA |
| Yes | 2 | 4 |  |
| Smoking |  |  |  |
| Never | 0 | 0 | 0 |
| Former | 0 | 1 | 0 |
| Current | 1 | 4 | 1.5 |
| Alcohol | NA |  |  |
| Abstain |  | 0 | 0 |
| Light-moderate |  | -3 | -1 |

| SUPPLEMENTARY TABLE 2 (continued) | |  |  |
| --- | --- | --- | --- |
|  | **CogD points** | **ANU-ADRI points** | **Modified-LIBRA** |
| Social engagement | NA |  | NA |
| Lowest |  | 6 |  |
| Below the median |  | 4 |  |
| Above the median |  | 1 |  |
| Highest |  | 0 |  |
| Loneliness |  | NA | NA |
| Yes | 2 |  |  |
| Physical activity |  |  |  |
| Lowest | 0 | 0 | 1.1 |
| Moderate | -3 | -2 | 0 |
| Vigorous | -3 | -3 | 0 |
| Cognitive activity |  |  |  |
| Lowest | 0 | 0 | 0 |
| Medium | -4 | -6 | 0 |
| Highest | -5 | -7 | -3.2 |
| Fish intake |  |  |  |
| <0.25 per week | NA | 0 | NA |
| 0.25-2 per week | NA | -3 | NA |
| 2-4 per week | NA | -4 | NA |
| >4 per week | NA | -5 | NA |
| Fish serves per week | -0.25 | NA | -1.7 |
| Medi-diet | NA | NA | -1.7 |
| Hypertension (<=65 Years) |  | NA |  |
| Yes | 1 |  | 1.6 |
| Stroke |  | NA | NA |
| Yes | 2 |  |  |
| Atrial fibrillation (>65 Years) |  |  |  |
| Yes | 2 | NA | NA |
| Insomnia |  |  |  |
| Yes | 2 | NA | NA |
| CHD |  |  |  |
| Yes | NA | NA | 1 |
| Renal dysfunction | NA | NA | 1.1 |
| Pesticides exposure |  |  |  |
| Yes | NA | 2 | NA |

Abbreviations: CogD, Cognitive Health and Dementia Risk Index; ANU-ADRI, Australian National University–Alzheimer Disease Risk Index; Modified-LIBRA, modified LIfestyle for BRAin health; NA: Not included in the tool; TBI, Traumatic brain injury; CHD, Coronary Heart Disease.

REFERENCES

1. Huque, M.H., et al., CogDrisk, ANU-ADRI, CAIDE, and LIBRA Risk Scores for Estimating Dementia Risk. JAMA Netw Open, 2023. 6(8): p. e2331460, doi:10.1001/jamanetworkopen.2023.31460

SUPPLEMENTARY TABLE 3 Baseline characteristics of included and excluded participants.

| Variables | Total population (n = 11652) | Excluded population  (n = 8690) | Included population  (n = 2962) | **χ^2^/t** | p-value |
| --- | --- | --- | --- | --- | --- |
| Age (years) (mean, SD) | 72.15 (6.14) | 71.72 (6.33) | 73.41 (5.34) | -14.17 | < 0.001 |
| Missing | 4.00 (0.03) | 3.00 (0.03) | 1.00 (0.03) |  |  |
| Education (years) (mean, SD) | 7.56 (5.29) | 7.88 (5.12) | 6.62 (5.66) | 10.73 | < 0.001 |
| Missing | 3.00 (0.03) | 3.00 (0.03) | 0.00 (0.00) |  |  |
| Living arrangement (%) |  |  |  | 158.01 | < 0.001 |
| Living alone | 1696 (14.56) | 1108 (12.75) | 588 (19.85) |  |  |
| Living with friends or caregivers | 147 (1.26) | 120 (1.38) | 27 (0.91) |  |  |
| Living with spouse only | 6515 (55.91) | 5118 (58.9) | 1397 (47.16) |  |  |
| Generations living together | 1892 (16.24) | 1321 (15.2) | 571 (19.28) |  |  |
| Missing | 1402 (12.03) | 1023 (11.77) | 379 (12.8) |  |  |
| Medical insurance |  |  |  | 43.84 | < 0.001 |
| No insurance | 739 (6.34) | 395 (4.55) | 344 (11.61) |  |  |
| Urban and rural residents’ basic medical insurance | 2211 (18.98) | 1220 (14.04) | 991 (33.46) |  |  |
| Employees’ basic medical insurance | 2230 (19.14) | 1419 (16.33) | 811 (27.38) |  |  |
| Commercial medical insurance | 22 (0.19) | 16 (0.18) | 6 (0.20) |  |  |
| Missing | 6450 (55.35) | 5640 (64.9) | 810 (27.35) |  |  |
| Income satisfaction (yes, %) | 6887 (59.11) | 5203 (59.87) | 1684 (56.85) | 27.03 | < 0.001 |
| Missing | 1642 (14.09) | 1282 (14.75) | 360 (12.15) |  |  |
| Familial emotional support (yes, %) | 3449 (29.6) | 1972 (22.69) | 1477 (49.86) | 0.09 | < 0.001 |
| Missing | 7552 (64.81) | 6341 (72.97) | 1211 (40.88) |  |  |
| Alive siblings (yes, %) | 6451 (55.36) | 4123 (47.45) | 2328 (78.6) | 682.02 | < 0.001 |
| Missing | 920 (7.90) | 852 (9.80) | 68 (2.30) |  |  |

| SUPPLEMENTARY TABLE 3 (continued) |  |  |  |  |  | |
| --- | --- | --- | --- | --- | --- | --- |
| Variables | Total population (n = 11652) | Excluded population  (n = 8690) | Included population  (n = 2962) | χ^2^/t | p-value | |
| Number of close friends (%) |  |  |  | 60.55 | < 0.001 | |
| None | 2457 (21.09) | 1688 (19.42) | 769 (25.96) |  |  | |
| 1 - 2 | 1921 (16.49) | 1369 (15.75) | 552 (18.64) |  |  | |
| 3 - 5 | 3088 (26.50) | 2289 (26.34) | 799 (26.98) |  |  | |
| 6 or More | 3167 (27.18) | 2455 (28.25) | 712 (24.04) |  |  | |
| Missing | 1019 (8.75) | 889 (10.23) | 130 (4.39) |  |  | |
| Height (cm) (mean (SD)) | 159.46 (8.83) | 159.86 (8.81) | 158.43 (8.79) | 7.38 | < 0.001 | |
| Missing | 1371.00 (11.77) | 1262.00 (14.52) | 109.00 (3.68) |  |  | |
| Weight (kg) (mean (SD)) | 60.53 (10.92) | 60.85 (10.97) | 59.7 (10.76) | 4.81 | < 0.001 | |
| Missing | 1379.00 (11.83) | 1249.00 (14.37) | 130.00 (4.39) |  |  | |
| Waistline (cm) (mean (SD)) | 86.62 (9.85) | 86.42 (9.87) | 87.22 (9.77) | -3.59 | 0.508 | |
| Missing | 1458.00 (12.51) | 1079.00 (12.42) | 379.00 (12.80) |  |  | |
| Right-hand grip strength (kg) (mean (SD)) | 22.24 (16.96) | 22.81 (20.15) | 21.43 (10.89) | 3.12 | < 0.001 | |
| Missing | 6618.00 (56.80) | 5735.00 (66.00) | 883.00 (29.81) |  |  | |
| Heart rate (mean (SD)) | 74.03 (13.73) | 73.94 (10.80) | 74.17 (17.40) | -0.54 | < 0.001 | |
| Missing | 6278.00 (53.88) | 5395.00 (62.08) | 883.00 (29.810) |  |  | |
| Hypertension (yes, %) | 6071 (52.10) | 4403 (50.67) | 1668 (56.31) | 30.14 | < 0.001 | |
| Missing | 125 (1.07) | 86 (0.99) | 39 (1.32) |  |  | |
| Anemia (yes, %) | 452 (3.88) | 362 (4.17) | 90 (3.04) | 3.04 | < 0.001 | |
| Missing | 4135 (35.49) | 2438 (28.06) | 1697 (57.29) |  |  |  |
| Head trauma (yes, %) | 745 (6.39) | 596 (6.86) | 149 (5.03) | 13.41 | < 0.001 |  |
| Missing | 426 (3.66) | 353 (4.06) | 73 (2.46) |  |  |  |

| SUPPLEMENTARY TABLE 3 (continued) |  |  |  |  |  |
| --- | --- | --- | --- | --- | --- |
| Variables | Total population (n = 11652) | Excluded population  (n = 8690) | Included population  (n = 2962) | χ^2^/t | p-value |
| Facial infection (yes, %) | 1044 (8.96) | 821 (9.45) | 223 (7.53) | 12.04 | < 0.001 |
| Missing | 507 (4.35) | 430 (4.95) | 77 (2.60) |  |  |
| Thyroid dysfunction (yes, %) | 688 (5.90) | 524 (6.03) | 164 (5.54) | 3.25 | < 0.001 |
| Missing | 932 (8.00) | 849 (9.77) | 83 (2.80) |  |  |
| Cervical spondylosis (yes, %) | 3506 (30.09) | 2624 (30.2) | 882 (29.78) | 0.92 | < 0.001 |
| Missing | 424 (3.64) | 354 (4.07) | 70 (2.36) |  |  |
| Depression symptoms (yes, %) | 32 (0.27) | 14 (0.16) | 18 (0.61) | 2.68 | < 0.001 |
| Missing | 5809 (49.85) | 5214 (60) | 595 (20.09) |  |  |
| Parkinson’s disease (yes, %) | 139 (1.19) | 76 (0.87) | 63 (2.13) | 2.00 | < 0.001 |
| Missing | 5355 (45.96) | 4860 (55.93) | 495 (16.71) |  |  |
| Family history of diabetes (yes, %) | 1332 (11.43) | 984 (11.32) | 348 (11.75) | 9.21 | < 0.001 |
| Missing | 3690 (31.67) | 3087 (35.52) | 603 (20.36) |  |  |
| Family history of stroke (yes, %) | 860 (7.38) | 647 (7.45) | 213 (7.19) | 11.21 | < 0.001 |
| Missing | 3757 (32.24) | 3144 (36.18) | 613 (20.7) |  |  |
| Fish consumption (yes, %) | 9862 (84.64) | 7261 (83.56) | 2601 (87.81) | 8.20 | < 0.001 |
| Missing | 854 (7.33) | 781 (8.99) | 73 (2.46) |  |  |
| Fruit consumption (yes, %) | 9922 (85.15) | 7299 (83.99) | 2623 (88.56) | 6.56 | < 0.001 |
| Missing | 865 (7.42) | 790 (9.09) | 75 (2.53) |  |  |
| Eating behaviors (%) |  |  |  | 6.88 | < 0.001 |
| Not special | 5812 (49.88) | 3532 (40.64) | 2280 (76.98) |  |  |
| Skipping breakfast | 128 (1.10) | 81 (0.93) | 47 (1.59) |  |  |
| Having afternoon tea | 95 (0.82) | 65 (0.75) | 30 (1.01) |  |  |
| Having midnight snacks | 92 (0.79) | 60 (0.69) | 32 (1.08) |  |  |

| SUPPLEMENTARY TABLE 3 (continued) |  |  |  |  |  |
| --- | --- | --- | --- | --- | --- |
| Variables | Total population (n = 11652) | Excluded population  (n = 8690) | Included population  (n = 2962) | χ^2^/t | p-value |
| Eating in the middle of the night | 4 (0.03) | 3 (0.03) | 1 (0.03) |  |  |
| Skipping lunch | 48 (0.41) | 29 (0.33) | 19 (0.64) |  |  |
| Skipping dinner | 52 (0.45) | 27 (0.31) | 25 (0.84) |  |  |
| Missing | 5421 (46.52) | 4893 (56.31) | 528 (17.83) |  |  |
| Cognitive activity (yes, %) | 6092 (52.28) | 4760 (54.78) | 1332 (44.97) | 102.46 | < 0.001 |
| Missing | 457 (3.92) | 372 (4.28) | 85 (2.87) |  |  |
| Nighttime awakenings (%) |  |  |  | 5.07 | < 0.001 |
| None | 1472 (12.63) | 859 (9.88) | 613 (20.70) |  |  |
| < 1 time/week | 1145 (9.83) | 649 (7.47) | 496 (16.75) |  |  |
| 1 - 2 times/week | 638 (5.48) | 349 (4.02) | 289 (9.76) |  |  |
| ≥ 3 times/week | 81 (0.70) | 39 (0.45) | 42 (1.42) |  |  |
| Missing | 8316 (71.37) | 6794 (78.18) | 1522 (51.38) |  |  |
| Hearing acuity (%) |  |  |  | 452.73 | < 0.001 |
| No change | 6035 (51.79) | 4959 (57.07) | 1076 (36.33) |  |  |
| Slight dullness | 1806 (15.5) | 1097 (12.62) | 709 (23.94) |  |  |
| Significant dullness | 418 (3.59) | 234 (2.69) | 184 (6.21) |  |  |
| Loss | 11 (0.09) | 6 (0.07) | 5 (0.17) |  |  |
| Missing | 3382 (29.03) | 2394 (27.55) | 988 (33.36) |  |  |
| Sense of smell (%) |  |  |  | 179.99 | < 0.001 |
| No change | 7105 (60.98) | 5583 (64.25) | 1522 (51.38) |  |  |
| Slight dullness | 926 (7.95) | 565 (6.5) | 361 (12.19) |  |  |
| Significant dullness | 138 (1.18) | 86 (0.99) | 52 (1.76) |  |  |
| Loss | 42 (0.36) | 18 (0.21) | 24 (0.81) |  |  |

| SUPPLEMENTARY TABLE 3 (continued) |  |  |  |  |  |
| --- | --- | --- | --- | --- | --- |
| Variables | Total population (n = 11652) | Excluded population  (n = 8690) | Included population  (n = 2962) | χ^2^/t | p-value |
| Missing | 3441 (29.53) | 2438 (28.06) | 1003 (33.86) |  |  |
| Vision acuity (%) |  |  |  | 18.03 | < 0.001 |
| No change | 1061 (9.11) | 684 (7.87) | 377 (12.73) |  |  |
| Slight dullness | 2569 (22.05) | 1506 (17.33) | 1063 (35.89) |  |  |
| Significant dullness | 863 (7.41) | 491 (5.65) | 372 (12.56) |  |  |
| Loss | 14 (0.12) | 12 (0.14) | 2 (0.07) |  |  |
| Missing | 7145 (61.32) | 5997 (69.01) | 1148 (38.76) |  |  |
| Walking pace (slower, %) | 3533 (30.32) | 2730 (31.42) | 803 (27.11) | 38.97 | < 0.001 |
| Missing | 1775 (15.23) | 1426 (16.41) | 349 (11.78) |  |  |
| Gait stability (yes, %) | 3652 (31.34) | 2071 (23.83) | 1581 (53.38) | 5.65 | < 0.001 |
| Missing | 7373 (63.28) | 6296 (72.45) | 1077 (36.36) |  |  |
| Defecation habit (%) |  |  |  | 10.84 | < 0.001 |
| Morning | 2585 (22.19) | 1438 (16.55) | 1147 (38.72) |  |  |
| Afternoon | 105 (0.90) | 54 (0.62) | 51 (1.72) |  |  |
| Night | 100 (0.86) | 48 (0.55) | 52 (1.76) |  |  |
| Irregular | 1071 (9.19) | 537 (6.18) | 534 (18.03) |  |  |
| Missing | 7791 (66.86) | 6613 (76.1) | 1178 (39.77) |  |  |
| Defecation duration (minutes) (mean (SD)) | 8.09 (7.81) | 7.88 (8.05) | 8.35 (7.54) | -1.86 | < 0.001 |
| Missing | 7867.00 (67.52) | 6649.00 (76.51) | 1218.00 (41.12) |  |  |
| Memory concerns (yes, %) | 3308 (28.39) | 2252 (25.91) | 1056 (35.65) | 14.72 | < 0.001 |
| Missing | 3767 (32.33) | 3138 (36.11) | 629 (21.24) |  |  |
|  |  |  |  |  |  |

| SUPPLEMENTARY TABLE 3 (continued) |  |  |  |  |  |
| --- | --- | --- | --- | --- | --- |
| Variables | Total population (n = 11652) | Excluded population  (n = 8690) | Included population  (n = 2962) | χ^2^/t | p-value |
| Having worse memory than peers (yes, %) | 2105 (18.07) | 1430 (16.46) | 675 (22.79) | 6.81 | < 0.001 |
| Missing | 4127 (35.42) | 3410 (39.24) | 717 (24.21) |  |  |
| Seeing a doctor due to memory concerns (yes, %) | 181 (1.55) | 124 (1.43) | 57 (1.92) | 0.29 | < 0.001 |
| Missing | 4500 (38.62) | 3642 (41.91) | 858 (28.97) |  |  |

SUPPLEMENTARY TABLE 4 Model performances for predicting incident cognitive impairment in the training dataset.

| **Models** | **AUC (95% CI)** | **Accuracy (95% CI)** | **Sensitivity (95% CI)** | **Specificity (95% CI)** | **PPV (95% CI)** | **NPV (95% CI)** |
| --- | --- | --- | --- | --- | --- | --- |
| **HMACS, thirty-eight key features after LASSO selection** | | | | | | |
| LR | 0.91 (0.89, 0.92) | 0.85 (0.83, 0.86) | 0.80 (0.74, 0.85) | 0.85 (0.84, 0.87) | 0.37 (0.33, 0.42) | 0.98 (0.97, 0.98) |
| KNN | 1.00 (1.00, 1.00) | 1.00 (1.00, 1.00) | 1.00 (0.98, 1.00) | 1.00 (1.00, 1.00) | 1.00 (0.98, 1.00) | 1.00 (1.00, 1.00) |
| DT | 0.50 (0.50, 0.50) | 0.90 (0.89, 0.92) | 0.00 (0.00, 0.02) | 1.00 (1.00, 1.00) | - | 0.90 (0.89, 0.92) |
| RF | 1.00 (1.00, 1.00) | 0.97 (0.96, 0.97) | 0.65 (0.58, 0.71) | 1.00 (1.00, 1.00) | 1.00 (0.97, 1.00) | 0.96 (0.95, 0.97) |
| XGB | 0.94 (0.93, 0.95) | 0.93 (0.92, 0.94) | 0.33 (0.27, 0.40) | 0.99 (0.99, 1.00) | 0.84 (0.74, 0.90) | 0.93 (0.92, 0.94) |
| LGBM | 0.99 (0.99, 1.00) | 0.97 (0.96, 0.97) | 0.67 (0.61, 0.73) | 1.00 (0.99, 1.00) | 0.96 (0.91, 0.98) | 0.97 (0.96, 0.97) |
| SVM | 0.94 (0.93, 0.96) | 0.93 (0.91, 0.94) | 0.25 (0.19, 0.31) | 1.00 (1.00, 1.00) | 0.96 (0.87, 0.99) | 0.93 (0.91, 0.94) |
| NNET | 0.91 (0.89, 0.93) | 0.94 (0.93, 0.95) | 0.75 (0.68, 0.80) | 0.96 (0.95, 0.97) | 0.67 (0.61, 0.73) | 0.97 (0.96, 0.98) |

Abbreviation: AUC, area under receiver operator characteristic curve; CI, confidence interval. PPV, positive predictive value; NPV, negative predictive value; HMACS, Hubei Memory and Aging Cohort Study; LASSO, Least Absolute Shrinkage and Selection Operator; LR, Logistic Regression; DT, Decision Tree; KNN, K-Nearest Neighbors; RF, Random Forest; XGB, eXtreme Gradient Boosting; LGBM, Light Gradient Boosting Machine; SVM, Support Vector Machine; NNET, Artificial Neural Network

SUPPLEMENTARY TABLE 5 Model performances for predicting incident cognitive impairment using the simplified model in the training dataset.

| **Models** | **AUC (95% CI)** | **Accuracy (95% CI)** | **Sensitivity (95% CI)** | **Specificity (95% CI)** | **PPV (95% CI)** | **NPV (95% CI)** |
| --- | --- | --- | --- | --- | --- | --- |
| **HMACS, fifteen key features after LASSO selection** | | | | | | |
| LR | 0.86 (0.84, 0.88) | 0.81 (0.80, 0.83) | 0.75 (0.69, 0.79) | 0.82 (0.80, 0.83) | 0.31 (0.28, 0.34) | 0.97 (0.96, 0.97) |
| KNN | 1.00 (1.00, 1.00) | 0.99 (0.99, 1.00) | 0.94 (0.91, 0.96) | 1.00 (1.00, 1.00) | 0.99 (0.97, 1.00) | 0.99 (0.99, 1.00) |
| DT | 0.50 (0.50, 0.50) | 0.90 (0.89, 0.91) | 0.00 (0.00, 0.01) | 1.00 (1.00, 1.00) | - | 0.90 (0.89, 0.91) |
| RF | 1.00 (1.00, 1.00) | 0.95 (0.94, 0.96) | 0.47 (0.42, 0.53) | 1.00 (1.00, 1.00) | 1.00 (0.97, 1.00) | 0.95 (0.94, 0.95) |
| XGB | 0.92 (0.91, 0.94) | 0.92 (0.91, 0.93) | 0.20 (0.15, 0.25) | 1.00 (0.99, 1.00) | 0.88 (0.78, 0.94) | 0.92 (0.91, 0.93) |
| LGBM | 0.95 (0.94, 0.96) | 0.92 (0.91, 0.93) | 0.27 (0.22, 0.32) | 0.99 (0.99, 1.00) | 0.82 (0.73, 0.89) | 0.93 (0.92, 0.93) |
| SVM | 0.96 (0.95, 0.97) | 0.93 (0.92, 0.94) | 0.27 (0.22, 0.32) | 1.00 (1.00, 1.00) | 0.99 (0.93, 1.00) | 0.93 (0.92, 0.94) |
| NNET | 0.88 (0.86, 0.90) | 0.92 (0.91, 0.93) | 0.27 (0.22, 0.32) | 0.99 (0.98, 0.99) | 0.72 (0.63, 0.79) | 0.93 (0.92, 0.93) |

Abbreviation: AUC, area under the receiver operator characteristic curve; CI, confidence interval. PPV, positive predictive value; NPV, negative predictive value; HMACS, Hubei Memory and Aging Cohort Study; LASSO, Least Absolute Shrinkage and Selection Operator; LR, Logistic Regression; DT, Decision Tree; KNN, K-Nearest Neighbors; RF, Random Forest; XGB, eXtreme Gradient Boosting; LGBM, Light Gradient Boosting Machine; SVM, Support Vector Machine; NNET, Artificial Neural Network

SUPPLEMENTARY TABLE 6 Model performances for predicting incident cognitive impairment using the simplified model in the test dataset.

| **Models** | **AUC (95% CI)** | **Accuracy (95% CI)** | **Sensitivity (95% CI)** | **Specificity (95% CI)** | **PPV (95% CI)** | **NPV (95% CI)** |
| --- | --- | --- | --- | --- | --- | --- |
| **CLHLS, fifteen key features after LASSO selection** | | | | | | |
| LR | 0.64 (0.62, 0.67) | 0.33 (0.32, 0.34) | 0.85 (0.83, 0.88) | 0.26 (0.25, 0.28) | 0.13 (0.12, 0.14) | 0.93 (0.92, 0.95) |
| KNN | 0.59 (0.57, 0.60) | 0.75 (0.74, 0.76) | 0.32 (0.29, 0.36) | 0.81 (0.80, 0.82) | 0.18 (0.16, 0.20) | 0.90 (0.90, 0.91) |
| DT | 0.50 (0.50, 0.50) | 0.89 (0.88, 0.90) | 0.00 (0.00, 0.01) | 1.00 (1.00, 1.00) | - | 0.89 (0.88, 0.90) |
| RF | 0.63 (0.61, 0.65) | 0.35 (0.34, 0.36) | 0.86 (0.83, 0.88) | 0.29 (0.27, 0.30) | 0.13 (0.12, 0.14) | 0.94 (0.93, 0.95) |
| XGB | 0.62 (0.60, 0.64) | 0.38 (0.37, 0.39) | 0.80 (0.77, 0.83) | 0.32 (0.31, 0.34) | 0.13 (0.12, 0.14) | 0.93 (0.92, 0.94) |
| LGBM | 0.60 (0.58, 0.62) | 0.36 (0.35, 0.37) | 0.79 (0.76, 0.82) | 0.31 (0.30, 0.32) | 0.13 (0.12, 0.14) | 0.92 (0.91, 0.93) |
| SVM | 0.63 (0.61, 0.65) | 0.39 (0.38, 0.40) | 0.81 (0.79, 0.84) | 0.34 (0.33, 0.35) | 0.14 (0.13, 0.15) | 0.94 (0.92, 0.94) |
| NNET | 0.63 (0.61, 0.65) | 0.78 (0.77, 0.79) | 0.28 (0.25, 0.31) | 0.84 (0.83, 0.85) | 0.18 (0.16, 0.20) | 0.90 (0.89, 0.91) |

Abbreviation: AUC, area under the receiver operator characteristic curve; CI, confidence interval. PPV, positive predictive value; NPV, negative predictive value; CLHLS, Chinese Longitudinal Healthy Longevity Survey; LASSO, Least Absolute Shrinkage and Selection Operator; LR, Logistic Regression; DT, Decision Tree; KNN, K-Nearest Neighbors; RF, Random Forest; XGB, eXtreme Gradient Boosting; LGBM, Light Gradient Boosting Machine; SVM, Support Vector Machine; NNET, Artificial Neural Network.

**SUPPLEMENTARY TABLE 7 Comparison of the predictive performance between the present logistic regression model and reported dementia risk prediction models.**

| **Models** | **AUC (95% CI)** | **Accuracy (95% CI)** | **Sensitivity (95% CI)** | **Specificity (95% CI)** | **PPV (95% CI)** | **NPV (95% CI)** | **p-value** |
| --- | --- | --- | --- | --- | --- | --- | --- |
| LR | 0.86 (0.82, 0.89) | 0.81 (0.78, 0.83) | 0.78 (0.68, 0.85) | 0.81 (0.78, 0.84) | 0.32 (0.26, 0.38) | 0.97 (0.95, 0.98) |  |
| CogDisk | 0.76 (0.72, 0.80) | 0.60 (0.57, 0.63) | 0.85 (0.77, 0.91) | 0.57 (0.53, 0.60) | 0.19 (0.16, 0.23) | 0.97 (0.95, 0.98) | < 0.05 |
| ANU-ADRI | 0.75 (0.70, 0.80) | 0.74 (0.71, 0.77) | 0.65 (0.55, 0.74) | 0.75 (0.72, 0.78) | 0.24 (0.19, 0.30) | 0.95 (0.93, 0.96) | < 0.05 |
| Modified-LIBRA | 0.75 (0.70, 0.79) | 0.72 (0.69, 0.75) | 0.68 (0.59, 0.77) | 0.73 (0.70, 0.76) | 0.23 (0.19, 0.28) | 0.95 (0.93, 0.97) | < 0.05 |

Results were obtained from the predicted probabilities generated by the regression model (LR). Cut-off values were selected by maximizing the Youden index (YI = sensitivity + specificity − 1).

Abbreviations: AUC = Area under the ROC Curve; LR, Logistic Regression; CogDisk, Cognitive Health and Dementia Risk Index; ANU-ADRI, Australian National University–Alzheimer Disease Risk Index; Modified-LIBRA, modified LIfestyle for BRAin health.

**SUPPLEMENTARY TABLE 8 LR model variable coefficient estimation and significance test results table.**

| Predicting variables | Estimate | Std. Error | Z value | Pr(>\|z\|) |
| --- | --- | --- | --- | --- |
| (Intercept) | 1.97 | 2.96 | 0.66 | 0.507 |
| Age | 0.09 | 0.02 | 4.90 | < 0.001 |
| Education | -0.20 | 0.03 | (6.47) | < 0.001 |
| Living arrangement | -0.08 | 0.07 | (1.25) | 0.212 |
| Medical insurance | -0.20 | 0.15 | (1.32) | 0.188 |
| Income satisfaction | 0.39 | 0.19 | 2.02 | 0.044 |
| Familial emotional support | -0.16 | 0.30 | (0.55) | 0.585 |
| Alive siblings | -0.24 | 0.19 | (1.27) | 0.204 |
| Number of close friends | -0.09 | 0.09 | (1.05) | 0.292 |
| Height | -0.04 | 0.01 | (2.80) | 0.005 |
| Weight | -0.02 | 0.02 | (0.99) | 0.322 |
| Waistline | 0.02 | 0.01 | 1.65 | 0.100 |
| Right-hand grip strength | -0.07 | 0.02 | (4.37) | < 0.001 |
| Heart rate | -0.01 | 0.01 | (1.37) | 0.172 |
| Hypertension | 0.39 | 0.19 | 2.08 | 0.038 |
| Anemia | -0.37 | 0.30 | (1.25) | 0.210 |
| Head trauma | -0.54 | 0.41 | (1.31) | 0.191 |
| Facial infection | 0.30 | 0.33 | 0.92 | 0.358 |
| Thyroid dysfunction | -0.07 | 0.60 | (0.12) | 0.904 |
| Cervical Spondylosis | -0.18 | 0.20 | (0.94) | 0.347 |
| Depression symptoms | 1.53 | 0.91 | 1.69 | 0.091 |
| Parkinsons disease | 0.35 | 0.45 | 0.79 | 0.430 |
| Family history of diabetes | 0.30 | 0.28 | 1.05 | 0.294 |
| Family history of stroke | -0.45 | 0.35 | (1.30) | 0.195 |
| Fish consumption | -0.19 | 0.24 | (0.79) | 0.428 |
| Fruit consumption | -0.29 | 0.24 | (1.22) | 0.224 |
| Eating behaviors | -0.33 | 0.18 | (1.84) | 0.065 |
| Cognitive activity | -0.75 | 0.23 | (3.29) | 0.001 |
| Nighttime awakenings | -0.28 | 0.12 | (2.45) | 0.014 |
| Hearing acuity | -0.66 | 0.21 | (3.17) | 0.002 |
| Sense of smell | -0.14 | 0.26 | (0.54) | 0.588 |
| Vision acuity | 0.17 | 0.14 | 1.20 | 0.231 |
| Walking pace | -0.27 | 0.23 | (1.14) | 0.255 |
| Gait stability | -0.24 | 0.23 | (1.07) | 0.286 |
| Defecation habit | -0.15 | 0.07 | (2.10) | 0.036 |
| Defecation duration | 0.03 | 0.01 | 2.69 | 0.007 |
| Memory concerns | 0.17 | 0.19 | 0.91 | 0.365 |
| Having worse memory than peers | 0.73 | 0.20 | 3.74 | < 0.001 |
| Seeing a doctor due to memory concerns | -0.35 | 0.55 | -0.644 | 0.520 |

**
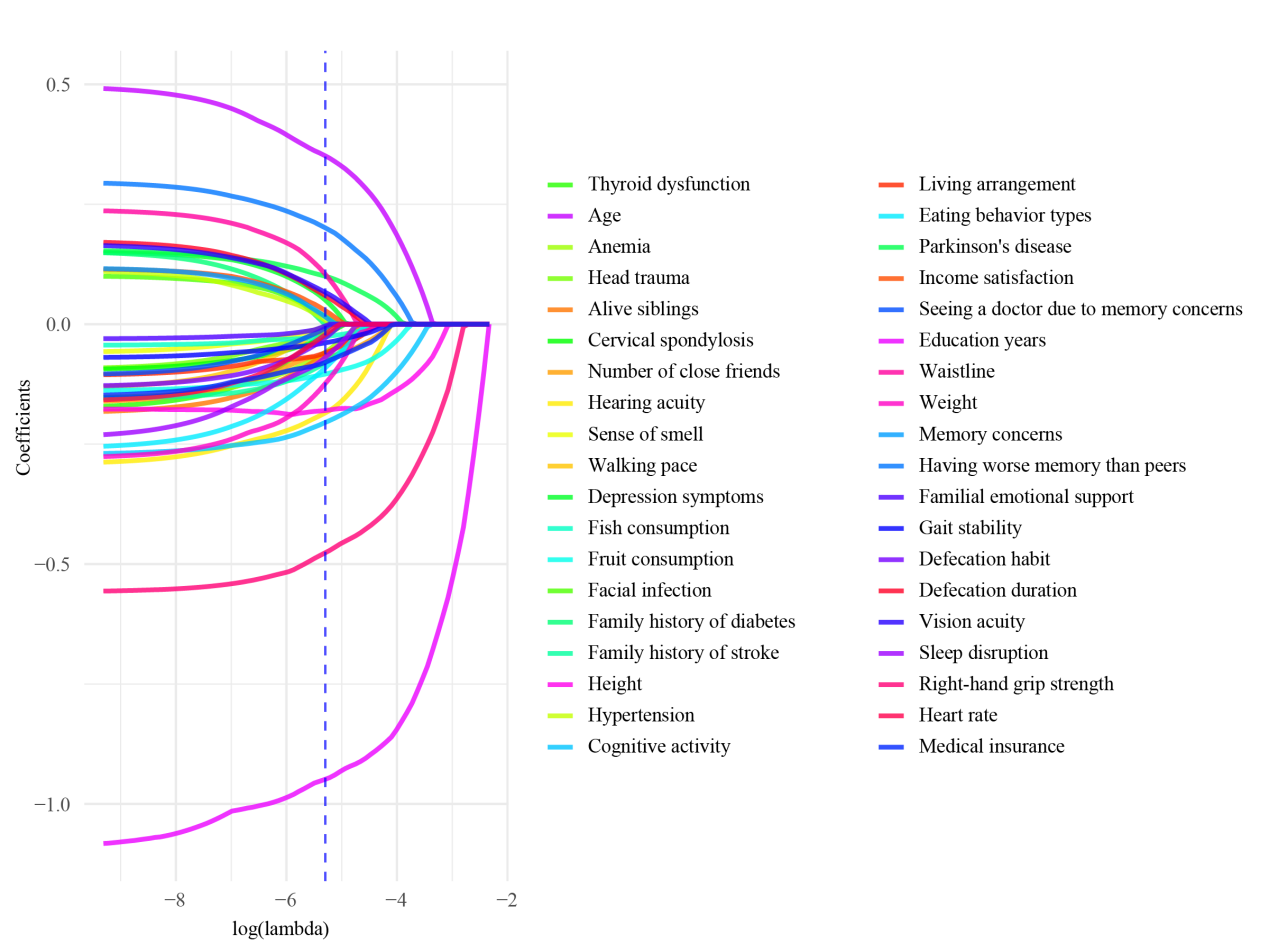
**

**SUPPLEMENTARY FIGURE 1 LASSO coefficient profiles of candidate predictors.**

**
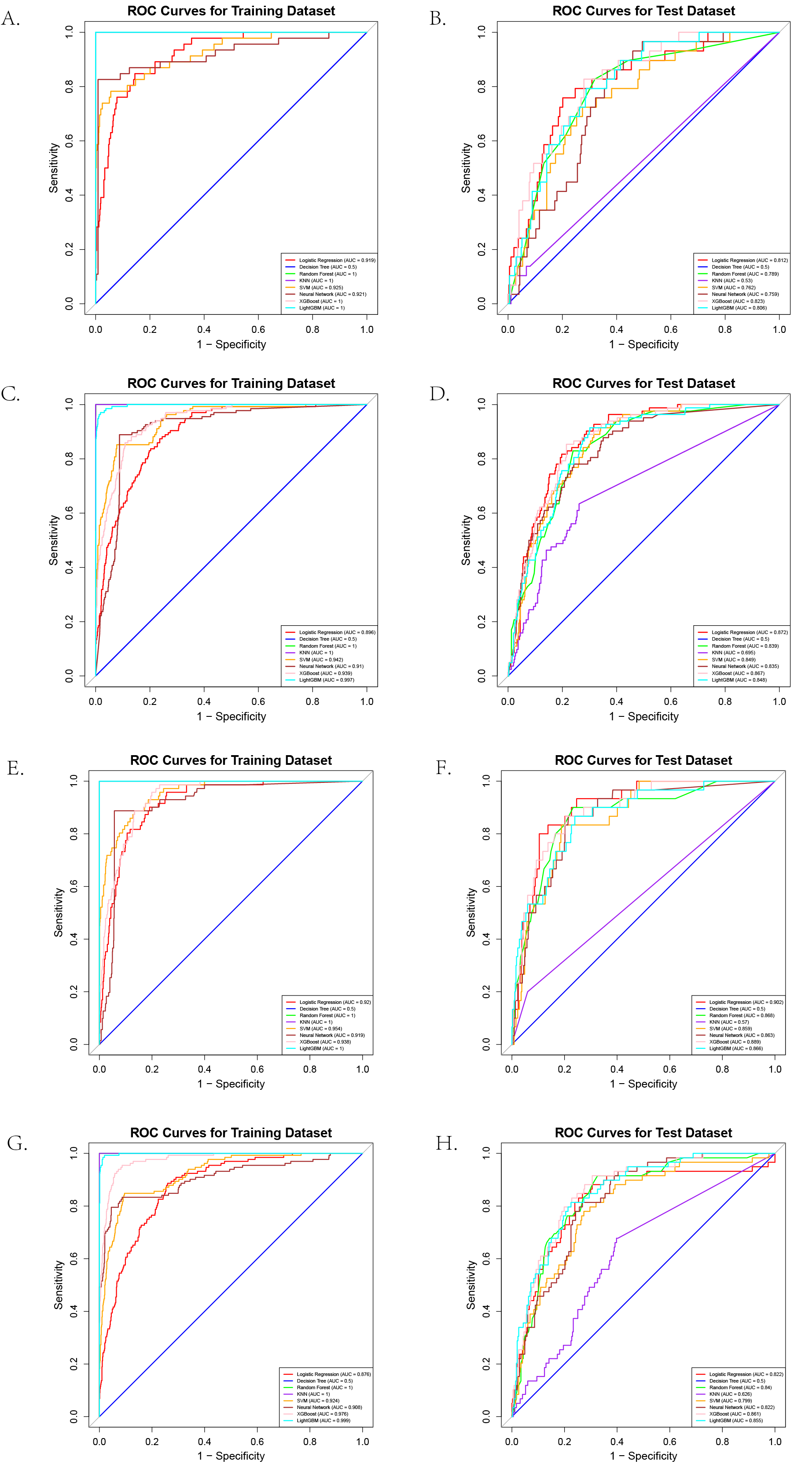
**

**SUPPLEMENTARY FIGURE 2 Sex- and age-stratified receiver operating characteristic curves of different machine learning models in the training and test sets.** A. The ROC curves of eight prediction models in the training dataset among male participants. B. The ROC curves of eight prediction models in the testing dataset among male participants. C. The ROC curves of eight prediction models in the training dataset among female participants. D. The ROC curves of eight prediction models in the testing dataset among female participants. E. ROC curves of eight prediction models in the training dataset among participants in the younger age group. F. ROC curves of eight prediction models in the testing dataset among participants in the younger age group. G. ROC curves of eight prediction models in the training dataset among participants in the older age group. H. ROC curves of eight prediction models in the testing dataset among participants in the older age group.

Abbreviation: HMACS, Hubei Memory and Aging Cohort Study; CLHLS, Chinese Longitudinal Healthy Longevity Survey; LR, Logistic Regression; DT, Decision Tree; KNN, K-Nearest Neighbors; RF, Random Forest; XGB, eXtreme Gradient Boosting; LGBM, Light Gradient Boosting Machine; SVM, Support Vector Machine; NNET, Artificial Neural Network.


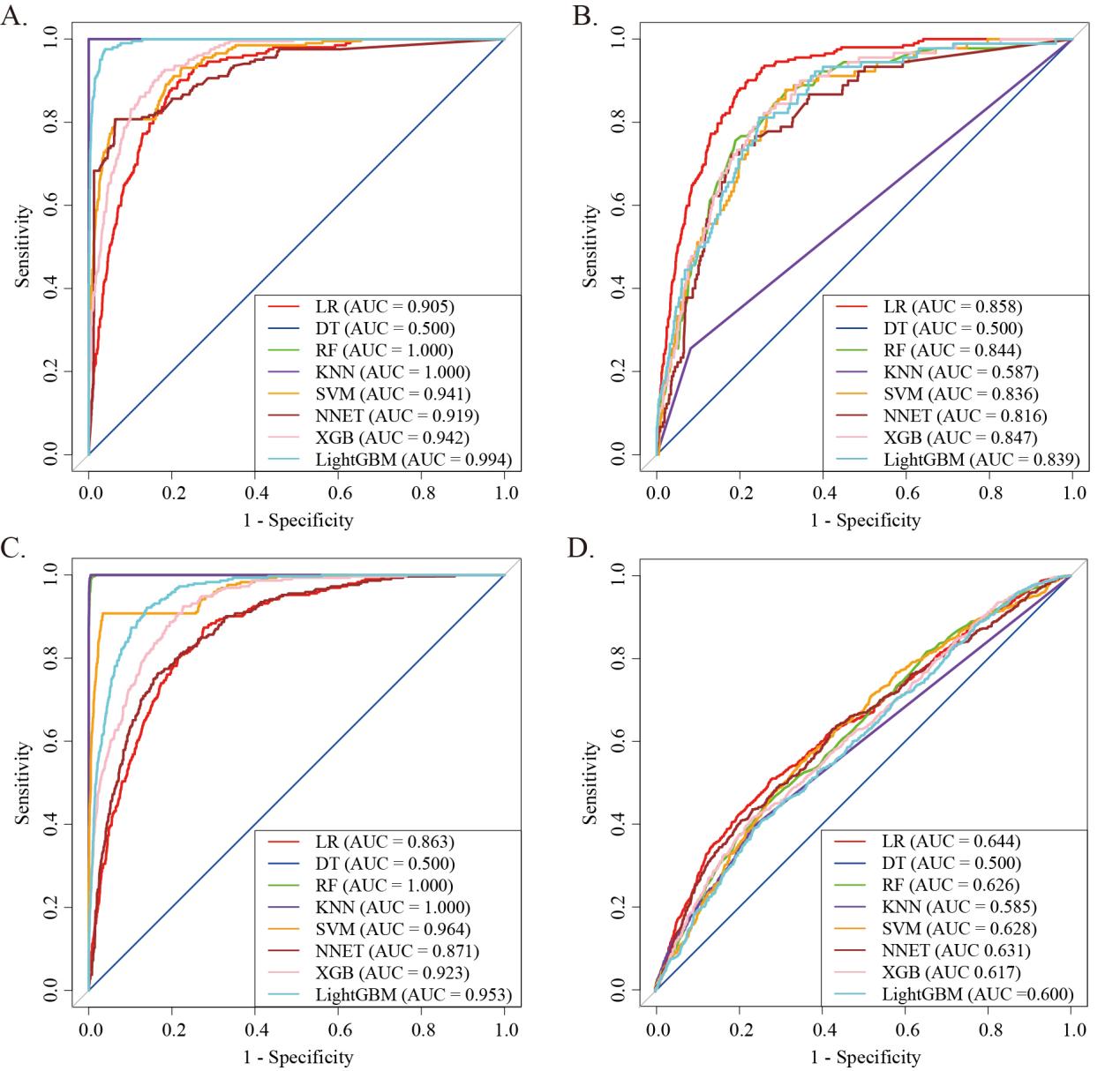
SUPPLEMENTARY FIGURE 3 ROC curves of machine learning models on the training and test datasets. A. The ROC curves of eight prediction models in the training dataset, where participants from HMACS were divided into training and testing datasets in a ratio of 7:3. B. The ROC curves of eight prediction models in the testing dataset, where the HMACS was divided into training and testing datasets in a ratio of 7:3. C. Due to the lack of some key variables in the CLHLS, the model was simplified using shared variables. The HMACS was used as the training dataset, and the CLHLS as the testing dataset. To verify whether eight different algorithms maintained performance after simplification. D. The ROC curves of the simplified model in the testing dataset.

Abbreviation: HMACS, Hubei Memory and Aging Cohort Study; CLHLS, Chinese Longitudinal Healthy Longevity Survey; LR, Logistic Regression; DT, Decision Tree; KNN, K-Nearest Neighbors; RF, Random Forest; XGB, eXtreme Gradient Boosting; LGBM, Light Gradient Boosting Machine; SVM, Support Vector Machine; NNET, Artificial Neural Network.
